# Avoiding gauge ambiguities in cavity quantum electrodynamics

**DOI:** 10.1038/s41598-021-83214-z

**Published:** 2021-02-19

**Authors:** Dominic M. Rouse, Brendon W. Lovett, Erik M. Gauger, Niclas Westerberg

**Affiliations:** 1 SUPA, School of Physics and Astronomy, University of St Andrews, St Andrews, KY16 9SS UK; 2 SUPA, Institute of Photonics and Quantum Sciences, Heriot-Watt University, Edinburgh, EH14 4AS UK; 3 School of Physics and Astronomy, University of Glasgow, Glasgow, G12 8QQ UK

**Keywords:** Optics and photonics, Physics

## Abstract

Systems of interacting charges and fields are ubiquitous in physics. Recently, it has been shown that Hamiltonians derived using different gauges can yield different physical results when matter degrees of freedom are truncated to a few low-lying energy eigenstates. This effect is particularly prominent in the ultra-strong coupling regime. Such ambiguities arise because transformations reshuffle the partition between light and matter degrees of freedom and so level truncation is a gauge dependent approximation. To avoid this gauge ambiguity, we redefine the electromagnetic fields in terms of potentials for which the resulting canonical momenta and Hamiltonian are explicitly unchanged by the gauge choice of this theory. Instead the light/matter partition is assigned by the intuitive choice of separating an electric field between displacement and polarisation contributions. This approach is an attractive choice in typical cavity quantum electrodynamics situations.

## Introduction

The gauge invariance of quantum electrodynamics (QED) is fundamental to the theory and can be used to greatly simplify calculations^1–8^. Of course, gauge invariance implies that physical observables are the same in all gauges despite superficial differences in the mathematics. However, it has recently been shown that the invariance is lost in the strong light/matter coupling regime if the matter degrees of freedom are treated as quantum systems with a fixed number of energy levels^8–14^, including the commonly used two-level truncation (2LT). At the origin of this is the role of gauge transformations (GTs) in deciding the partition between the light and matter degrees of freedom, even if the primary role of gauge freedom is to enforce Gauss’s law. Despite its long history^15–19^, this has led to new questions about which gauge most accurately describes the physics.

Two common gauge choices of non-relativistic QED ^9,10,14^ are the Coulomb gauge, which has the advantage of describing photons as purely transverse radiation modes, and the multipolar gauge, which is most useful when the leading order (dipole) terms are dominant in a multipole expansion of the fields^20–22^. Interestingly, within the 2LT the multipolar gauge is usually found to agree more closely with exact, gauge invariant calculations than the Coulomb gauge^9,10^. This has been attributed to the fact that in the Coulomb gauge the light/matter interaction strength scales with the transition frequency between the relevant matter levels, while in the multipolar gauge the coupling instead scales with the energy of the radiation mode. Therefore, transitions between well-separated matter levels can be non-negligible in the Coulomb gauge^9,10^. Further, Ref.^14^ suggested that the 2LT in the Coulomb gauge converts the local potential into a non-local one, which no longer only depends on position but now also on the gauge-dependent canonical momentum. This is further discussed in Refs.^23–25^ for a variety of physical settings.

The implication of these results is that the multipolar gauge is usually more accurate when the matter system is quantised and truncated to two levels. We should stress, however, that all gauges are equivalent and yield the same results if no approximations are made—a situation in which the so-called Power–Zienau–Woolley Hamiltonian is appropriate (found in for instance Ref. ^2^). Nonetheless, after approximations are made (such as a 2LT or a Born–Oppenheimer approximation) separate gauges can yield differing results, since energy levels have different meanings in different gauges. With regards to this, it has been emphasised^8,11^ that there exist a continuum of possible GTs, each with a unique light/matter partition and therefore also 2LT; depending on the physical setting, gauges other than the common choices can offer more accurate 2LTs^8,11,26^. Recently, it has also been reported that time-dependent light/matter couplings can lead to gauge ambiguities^27^.

**Figure 1:**
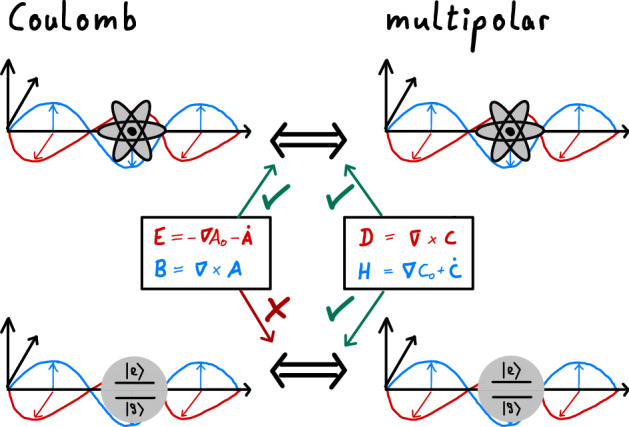
A schematic of the electromagnetic potentials in the conventional and new approaches. On the left, the electromagnetic fields are parametrised using conventional $\mathbf {A}$- and $A_0$-fields, respectively, whereas on the right, the $\mathbf {C}$- and $C_0$-fields are used. The various relations and equivalences between the gauges are also shown. In particular note that gauge ambiguities manifest when the matter levels are truncated using the $\mathbf {A}$-fields.

In this Article, we first review the conventional approach in section “Conventional approach”, after which we reformulate QED such that gauge ambiguities do not manifest, see Fig. 1, in section “New approach”. We thus separate gauge issues from the choice of light/matter partition, for which we now offer an alternative interpretation. Our reformulation builds on previous work on the dual representation of QED^28–33^. We then show that the dual representation recovers the multipolar-gauge Hamiltonian of the conventional theory when the light/matter partition is chosen appropriately. We also provide a physical explanation as to why this choice is optimal for systems typical of QED, e.g. dipoles in a cavity. Additionally, in section “Accuracy of two-level truncations”, we numerically compare the accuracy of 2LTs in different light/matter partitions for the example of dipoles in a cavity. The results are discussed in section “Discussion”. We provide further details, extensions to the model, and derivations in the Supplementary Material.

## Conventional approach

We first outline the conventional approach, and how gauge ambiguities arise in it (see Supplementary Material 1 for full mathematical details). Let us consider a generic system of charges $q_{\mu }$ at positions $\mathbf {r}_{\mu }$ described by a charge density $\rho$ and current density $\mathbf {J}$. Their dynamics are governed by the Maxwell equations and Lorentz force:

1a $$\begin{aligned}&\varvec{\nabla }\cdot \mathbf {B}(\mathbf {x})=0, \end{aligned}$$

1b $$\begin{aligned}&\varvec{\nabla }\times \mathbf {E}(\mathbf {x})=-\dot{\mathbf {B}}(\mathbf {x}), \end{aligned}$$

1c $$\begin{aligned}&\varvec{\nabla }\cdot \mathbf {E}(\mathbf {x})=\rho (\mathbf {x})/\varepsilon _0, \end{aligned}$$

1d $$\begin{aligned}&\varvec{\nabla }\times \mathbf {B}(\mathbf {x})=\mu _0\mathbf {J}(\mathbf {x})+\varepsilon _0\mu _0\dot{\mathbf {E}}(\mathbf {x}), \end{aligned}$$

1e $$\begin{aligned}&m_{\mu }\ddot{\mathbf {r}}_{\mu }=q_{\mu }\left[ \mathbf {E}(\mathbf {r}_{\mu })+\dot{\mathbf {r}}_{\mu }\times \mathbf {B}(\mathbf {r}_{\mu })\right] . \end{aligned}$$

Conventionally, the electric and magnetic fields are parametrised in terms of vector and scalar potentials $\mathbf {A}$ and $A_0$ as $\mathbf {E}= -\varvec{\nabla }A_0-\dot{\mathbf {A}}$ and $\mathbf {B} = \varvec{\nabla }\times \mathbf {A}$ respectively, leading immediately to Faraday’s law and sourceless magnetic fields ^34^. The remaining equations are derived by minimizing the action of the *minimal-coupling Lagrangian*^2,4,5,7,8^

2 $$\begin{aligned} L_m=\sum _{\mu }\frac{1}{2}m_{\mu }\dot{\mathbf {r}}^2_{\mu }+\int d^3x\ \mathcal {L}_m(\mathbf {x}), \end{aligned}$$

with the Lagrange density

3 $$\begin{aligned} \mathcal {L}_m(\mathbf {x}) = \frac{\varepsilon _0}{2}\left[ \mathbf {E}^2 - c^2\mathbf {B}^2\right] +\left[ \mathbf {J}\cdot \mathbf {A}(\mathbf {x})-\rho A_0\right] , \end{aligned}$$

where the mechanical degrees of freedom are $A_0$, $\mathbf {A}$ and $\mathbf {r}_\mu$, respectively. The physical fields are unchanged by the introduction of a scalar field

4 $$\begin{aligned} \chi (\mathbf {x})=\int d^3x^{\prime }\ \tilde{\chi }(\mathbf {x},\mathbf {x}^{\prime }), \end{aligned}$$

so long as

5a $$\begin{aligned}&A_0^{\prime }(\mathbf {x})=A_0(\mathbf {x})+\dot{\chi }(\mathbf {x}), \end{aligned}$$

5b $$\begin{aligned}&\mathbf {A}^{\prime }(\mathbf {x})=\mathbf {A}(\mathbf {x})-\varvec{\nabla }\chi (\mathbf {x}), \end{aligned}$$

where a primed variable indicates one transformed by the $\chi$-field. Under this transformation the Lagrangian $L^{\prime }_m$ has a modified Lagrange density given by

6 $$\begin{aligned} {\mathcal {L}_m^{\prime }(\mathbf {x})}= \mathcal {L}_m(\mathbf {x})- \mathbf {J}(\mathbf {x})\cdot \varvec{\nabla }\chi (\mathbf {x})-\rho (\mathbf {x})\int d^3x^{\prime }\ \dot{\mathbf {A}}(\mathbf {x}^{\prime })\cdot \frac{\partial {\tilde{\chi }(\mathbf {x},\mathbf {x}^{\prime })}}{\partial {\mathbf {A}(\mathbf {x}^{\prime })}}. \end{aligned}$$

The canonical momenta of this arbitrary-gauge Lagrangian, $\mathbf {p}_{\mu }^{\prime }=\partial L^{\prime }_m/\partial \dot{\mathbf {r}}_{\mu }$ and ${{\varvec{\Pi }}}^{\prime }=\delta \mathcal {L}^{\prime }_m/\delta \dot{\mathbf {A}}$, can be found as^2^

7a $$\begin{aligned}&\mathbf {p}_{\mu }^{\prime }=m_{\mu }\dot{\mathbf {r}}_{\mu }+q_{\mu }\mathbf {A}(\mathbf {r}_{\mu })-q_{\mu }\varvec{\nabla }\chi (\mathbf {r}_{\mu }), \end{aligned}$$

7b $$\begin{aligned}&{{\varvec{\Pi }}}^{\prime }(\mathbf {x})=-\varepsilon _0\mathbf {E}(\mathbf {x})-\varvec{\phi }^{\prime }(\mathbf {x}),\end{aligned}$$

where

8 $$\begin{aligned} \varvec{\phi }^{\prime }(\mathbf {x})=\int d^3x^{\prime }\ \rho (\mathbf {x}^{\prime })\frac{\partial {\tilde{\chi }(\mathbf {x},\mathbf {x}^{\prime })}}{\partial {\mathbf {A}(\mathbf {x})}}. \end{aligned}$$

Importantly, $\mathbf {p}_{\mu }^{\prime }$ and ${{\varvec{\Pi }}}^{\prime }$ are explicitly gauge dependent and so correspond to different canonical momenta in every gauge^2,8^. After eliminating $A_0$ using the continuity equation, the arbitrary-gauge Hamiltonian is found as^2^

9 $$\begin{aligned} H^{\prime }=\sum _{\mu }\frac{1}{2m_{\mu }}\bigg [\mathbf {p}^{\prime }_{\mu } - q_{\mu }\mathbf {A}(\mathbf {r}_{\mu })+q_{\mu }\varvec{\nabla }\chi (\mathbf {r}_{\mu })\bigg ]^2+\int d^3x\ \left( \frac{1}{2\varepsilon _0}\left[ {{\varvec{\Pi }}}^{\prime }(\mathbf {x})+\varvec{\phi }^{\prime }(\mathbf {x})\right] ^2 + \frac{\mathbf {B}^2(\mathbf {x})}{2\mu _0}\right) . \end{aligned}$$

Gauge ambiguities can occur, particularly in the strong light/matter coupling regime, when approximations to the Hamiltonian are introduced. A prominent example of this is expressing the matter Hamiltonian using a truncated number of energy levels; this approximation has different meanings in each gauge. When quantizing, the gauge dependent classical momentum $\mathbf {p}^{\prime }_\mu$ is promoted to its quantum counterpart $\widehat{\mathbf {p}}^{\prime }_\mu$ (along with the position $\mathbf {r}_\mu \rightarrow \hat{\mathbf {r}}_\mu$). The truncation to $N+1$ discrete energy levels follows next for each charge:

10 $$\begin{aligned} \widehat{\mathbf {T}}^{\prime }_{\mu }\equiv \frac{\widehat{\mathbf {p}}^{\prime 2}_{\mu }}{2m_{\mu }}+\widehat{U}_{\mathrm {ext}}\left( \hat{\mathbf {r}}_\mu \right) \rightarrow \sum _{n=0}^N\epsilon _{n,\mu }\left| \epsilon _{n,\mu }^{\prime }\right\rangle \left\langle \epsilon _{n,\mu }^{\prime }\right| , \end{aligned}$$

where $\widehat{U}_{\mathrm {ext}}$ is the external electrostatic interaction binding the charges. Each ‘matter’ eigenstate in Eq. (10) refers to a different physical system in each gauge and so truncation means losing different information. Formally, only when $N\rightarrow \infty$ do all observables agree in different gauges, though for weak light/matter coupling a low-level truncation is usually sufficient for good agreement.

## New approach

The canonical momenta in the theory outlined above inherit their gauge-dependency from the minimal-coupling Lagrangian [Eq. (2)], as the vector and scalar potentials are only defined up to the scalar function $\chi$. To remove gauge ambiguities, we will therefore derive a theory which is described by a Lagrangian depending only on the physical fields.

The total charge and current densities of any system can be partitioned into *bound* and *free* contributions as $\rho =\rho _b+\rho _f$ and $\mathbf {J}=\mathbf {J}_b+\mathbf {J}_f$^34^. This naturally allows one to distinguish two contributions to the electric and magnetic fields: $\mathbf {E}=(\mathbf {D}-\mathbf {P})/\varepsilon _0$ and $\mathbf {B}=\mu _0(\mathbf {H}+\mathbf {M})$ with $\mathbf {P}$ and $\mathbf {M}$ being the polarisation and magnetisation fields. Our aim now is to parametrise the displacement and magnetic fields $\mathbf {D}$ and $\mathbf {H}$ using a dual vector potential $\mathbf {C}$ and scalar potential $C_0$ such that

11a $$\begin{aligned} \mathbf {D}(\mathbf {x})&=\varvec{\nabla }\times \mathbf {C}(\mathbf {x}), \end{aligned}$$

11b $$\begin{aligned} \mathbf {H}(\mathbf {x})&=\varvec{\nabla }C_0(\mathbf {x})+\dot{\mathbf {C}}(\mathbf {x}). \end{aligned}$$

This is the crucial point of this Article, and as we will show, it avoids gauge ambiguities in the formulation of cavity QED. The parametrization in terms of $\mathbf {C}$-fields relies on the absence of free currents $\mathbf {J}_f$, a common cavity QED setting ^8–11,14^. Other examples of defining the physical fields in this way can be found in^28–33^, although here we extend the formulation to include the magnetization field and therefore move beyond the standard electric dipole approximation.

The polarization field $\mathbf {P}$ and the magnetisation field $\mathbf {M}$ are sourced by the bound charge and currents, respectively:

12a $$\begin{aligned} \varvec{\nabla }\cdot \mathbf {P}\left( \mathbf {x},\mathbf {r}\right)&= -\rho _b\left( \mathbf {x},\mathbf {r}\right) , \end{aligned}$$

12b $$\begin{aligned} \varvec{\nabla }\times \mathbf {M}\left( \mathbf {x},\mathbf {r}\right)&= \mathbf {J}_b\left( \mathbf {x},\mathbf {r}\right) - \dot{\mathbf {P}}\left( \mathbf {x},\mathbf {r}\right) . \end{aligned}$$

We also note that Maxwell’s equations Eqs. (1c) and (1d) become:

13a $$\begin{aligned} \varvec{\nabla }\cdot \mathbf {D}(\mathbf {x})&= \rho _f(\mathbf {x}), \end{aligned}$$

13b $$\begin{aligned} \varvec{\nabla }\times \mathbf {H}(\mathbf {x})&= \mathbf {J}_f(\mathbf {x})+\dot{\mathbf {D}}(\mathbf {x}), \end{aligned}$$

when written in terms of the displacement field $\mathbf {D}$ and magnetic field $\mathbf {H}$. Note that interestingly, within this formalism Eqs. (1a)–(1b) and Eqs. (1c)–(1d) switch roles, as Eqs. (1a)–(1b) are dynamical equations for the $\mathbf {C}$-field with Eqs. (1c)–(1d) serving as the Bianchi identity, whereas the opposite is true for the $\mathbf {A}$-field.

We now specify a system to illustrate the theory, and for simplicity we will choose a single dipole formed of an electron at position $\mathbf {r}$ and a hole at the origin. The bound charge density and current of this dipole are described by $\rho _b\left( \mathbf {x},\mathbf {r}\right) = -e\delta (\mathbf {x}-\mathbf {r})+e\delta (\mathbf {x})$ and $\mathbf {J}_b\left( \mathbf {x},\mathbf {r}\right) = -e\dot{\mathbf {r}}\delta (\mathbf {x}-\mathbf {r})$, respectively. The theory is easily extended to more dipoles, and in Supplementary Material 2 we add a background ionic lattice which allows for phonon-mediated processes within the system. There are no free charges or currents ($\rho _f=\mathbf {J}_f=0$) and so a symmetry emerges when comparing Eq. (13) to Maxwell’s equations Eqs. (1a) and (1b)^20,28–34^. We will exploit this symmetry to parametrise the displacement and magnetic fields according to Eq. (11).

The restrictions on $\mathbf {P}$ and $\mathbf {M}$ given by Eq. (12) produce the correct bound charge density and current if^2,8,27^

14 $$\begin{aligned} \mathbf {P}\left( \mathbf {x},\mathbf {r}\right) =-e\int _0^1 d\lambda \ \mathbf {r} \delta (\mathbf {x}-\lambda \mathbf {r}), \end{aligned}$$

and

15 $$\begin{aligned} \mathbf {M}\left( \mathbf {x},\mathbf {r}\right) =-\dot{\mathbf {r}}\times \varvec{\theta }(\mathbf {x},\mathbf {r}), \end{aligned}$$

where

16 $$\begin{aligned} \varvec{\theta }(\mathbf {x},\mathbf {r})=-e\int _0^1 d\lambda \ \lambda \mathbf {r}\delta (\mathbf {x}-\lambda \mathbf {r}). \end{aligned}$$

However, these are not unique and Eq. (12) are also satisfied by $\mathbf {P}\rightarrow \widetilde{\mathbf {P}}=\mathbf {P}+\widetilde{\mathbf {P}}_V$ and $\mathbf {M}\rightarrow \widetilde{\mathbf {M}}=\mathbf {M}-\widetilde{\mathbf {M}}_V$ where

17a $$\begin{aligned}&\widetilde{\mathbf {P}}_V\left( \mathbf {x},\mathbf {r}\right) =\varvec{\nabla }\times \mathbf {V}\left( \mathbf {x},\mathbf {r}\right) , \end{aligned}$$

17b $$\begin{aligned}&\widetilde{\mathbf {M}}_V\left( \mathbf {x},\mathbf {r}\right) =\dot{\mathbf {V}}\left( \mathbf {x},\mathbf {r}\right) +\varvec{\nabla }V_0\left( \mathbf {x},\mathbf {r}\right) , \end{aligned}$$

for general fields $\mathbf {V}$ and $V_0$ and quantities dependent on these are denoted with a tilde. Such a transformation does not change the physics, but alters the light/matter partition. We emphasise that, in contrast, for the conventional $\mathbf {A}$-field theory the light/matter partition is encompassed by gauge freedom.

All that remains to complete the theory is to write a Lagrangian that reproduces the remaining Maxwell equations [Eqs. (1a) and (1b)], and Lorentz force equation [Eq. (1e)], when minimised with respect to the mechanical degrees of freedom $C_0$, $\mathbf {C}$ and $\mathbf {r}$ respectively. We find that the required Lagrangian is

18 $$\begin{aligned} L&=\frac{1}{2}m\dot{\mathbf {r}}^2+\int d^3x\ \frac{\varepsilon _0}{2}\left[ c^2\mathbf {B}^2(\mathbf {x})-\mathbf {E}^2(\mathbf {x})\right] \\&= \frac{1}{2}m\dot{\mathbf {r}}^2+\int d^3x\ \frac{\varepsilon _0}{2}\bigg [c^2\left( \dot{\mathbf {C}}+\varvec{\nabla }C_0 + \mathbf {M}\right) ^2-\left( \varvec{\nabla }\times \mathbf {C}-\mathbf {P}\right) ^2\bigg ], \end{aligned}$$

which for $\mathbf {M} \rightarrow \mathbf {0}$ agrees with Refs. ^33,64^ and we prove in Supplementary Material 3 that this Lagrangian satisfies all the necessary equations of motion.

Equations (11) are invariant under gauge transformations $C_0\rightarrow C_0+\dot{\xi }$ and $\mathbf {C}\rightarrow \mathbf {C}-\varvec{\nabla }\xi$ for any arbitrary scalar field $\xi$, but importantly so is the Lagrangian in Eq. (18). This is because the Lagrangian is written only in terms of the physical fields. Additionally, this means that the Lagrangian is invariant under the transformations in Eq. (17). It is also possible to verify this with a Lagrangian written in terms of the mechanical variables $C_0$, $\mathbf {C}$ and $\mathbf {r}$. For $\mathbf {B}$ and $\mathbf {E}$ to be invariant under this transformation, there must be an implicit change to the fields $\mathbf {C}$ and $C_0$, which we write explicitly as $\mathbf {D}\rightarrow \widetilde{\mathbf {D}}$ and $\mathbf {H}\rightarrow \widetilde{\mathbf {H}}$ where

19 $$\begin{aligned}&\widetilde{\mathbf {D}}(\mathbf {x})\equiv \varvec{\nabla }\times \widetilde{\mathbf {C}}(\mathbf {x})=\mathbf {E}(\mathbf {x})+\widetilde{\mathbf {P}}\left( \mathbf {x},\mathbf {r}\right) , \end{aligned}$$

20 $$\begin{aligned}&\widetilde{\mathbf {H}}(\mathbf {x})\equiv \dot{\widetilde{\mathbf {C}}}(\mathbf {x})+\nabla \widetilde{C}_0(\mathbf {x})=\mathbf {B}(\mathbf {x})-\widetilde{\mathbf {M}}\left( \mathbf {x},\mathbf {r}\right) . \end{aligned}$$

This is a direct consequence of the transformation in Eq. (17) changing the light/matter partition; a redistribution of the contributions of $\mathbf {D}^{\perp }$ and $\mathbf {P}^{\perp }$ to $\mathbf {E}^{\perp }$, and likewise $\mathbf {H}$ and $\mathbf {M}$ to $\mathbf {B}$. Note that we have here introduced the Helmholtz decomposition of a vector $\mathbf {W}=\mathbf {W}^{\parallel }+\mathbf {W}^{\perp }$ into parallel $\mathbf {W}^{\parallel }$ and perpendicular $\mathbf {W}^{\perp }$ components, satisfying $\varvec{\nabla }\times \mathbf {W^{\parallel }}=0$ and $\varvec{\nabla }\cdot \mathbf {W}^{\perp }=0$ respectively.

The gauge-invariant Lagrangian in Eq. (18) leads to the crucial result that the canonical momenta in the new theory are also no longer gauge dependent, although they do depend on the light/matter partition through $\mathbf {V}$ and $V_0$. We find that the canonical momenta are

21a $$\begin{aligned}&\widetilde{\mathbf {p}}=\frac{\partial {\widetilde{L}}}{\partial {\dot{\mathbf {r}}}}=m\dot{\mathbf {r}}-\varvec{\Phi }_0(\mathbf {r})-\widetilde{\varvec{\Phi }}(\mathbf {r}), \end{aligned}$$

21b $$\begin{aligned}&\widetilde{{{\varvec{\Pi }}}}(\mathbf {x})=\frac{\delta {\widetilde{\mathcal {L}}}}{\delta {\dot{\widetilde{\mathbf {C}}}(\mathbf {x})}}=\mathbf {B}(\mathbf {x}), \end{aligned}$$

where $$\varvec{\Phi }_0(\mathbf {r})=\int d^3x\ \varvec{\theta }(\mathbf {x},\mathbf {r})\times \mathbf {B}(\mathbf {x})$$ and

22 $$\begin{aligned} \widetilde{\varvec{\Phi }}(\mathbf {r})=\frac{\partial }{\partial {\dot{\mathbf {r}}}}\int d^3x\ \mathbf {B}(\mathbf {x})\cdot \widetilde{\mathbf {M}}_V\left( \mathbf {x},\mathbf {r}\right) . \end{aligned}$$

We derive Eq. (21) in Supplementary Material 4. We see that the field canonical momentum is always the magnetic field whilst the matter canonical momentum is dependent on the light/matter partition.

To derive the Hamiltonian, we must be able to invert Eq. (21a) to write $\dot{\mathbf {r}}$ as a function of $\mathbf {p}$. This puts a constraint on the allowed $\mathbf {V}$ and $V_0$ fields in the transformations in Eq. (17). Here we assume that this constraint is met which results in $\widetilde{\varvec{\Phi }}$ being independent of $\dot{\mathbf {r}}$, in which case we find that the Hamiltonian is

23 $$\begin{aligned} \widetilde{H}= \frac{1}{2m}\left[ \widetilde{\mathbf {p}}+\varvec{\Phi }_0(\mathbf {r})+\widetilde{\varvec{\Phi }}(\mathbf {r})\right] ^2+\widehat{U}_{\mathrm {ext}}+\int d^3x\ \left( \frac{\mathbf {B}^2(\mathbf {x})}{2\mu _0} + \frac{1}{2\varepsilon _0}\left[ \widetilde{\mathbf {D}}^{\perp }(\mathbf {x})-\widetilde{\mathbf {P}}(\mathbf {x})\right] ^2\right) , \end{aligned}$$

where we have introduced an external potential $\widehat{U}_{\mathrm {ext}}$. Equation (23) is derived explicitly in Supplementary Material 4 but follows the standard procedure. The gauge independence of Eq. (23) follows from the absence of magnetic monopoles, and as such the primary constraint $\varvec{\nabla }\cdot \mathbf {B} = 0$ can be satisfied without altering the light/matter partition. Note that, differently to $\mathbf {A}$-field theory, Gauss’s law can be enforced as an initial condition^33^. The constraint on inverting Eq. (21a) manifests as an additional term in Eq. (23) with the form

24 $$\begin{aligned} \left( 1-\dot{\mathbf {r}}\cdot \frac{\partial }{\partial {\dot{\mathbf {r}}}}\right) \int d^3x\ \mathbf {B}(\mathbf {x})\cdot \widetilde{\mathbf {M}}_V\left( \mathbf {x},\mathbf {r}\right) . \end{aligned}$$

This term vanishes when Eq. (21a) can be inverted, e.g. for $(\mathbf {V},V_0)=(\mathbf {0},0)$ and $(\mathbf {V},V_0)=(\frac{1}{2}\mathbf {r}\times \mathbf {x},0)$.

Before quantising the fields, we must choose a light/matter partition. In the conventional theory this requires a choice of gauge, however, the gauge choice here does not alter this partition. Instead, this freedom is encompassed in the choice of $\mathbf {P}^{\perp }$ and $\mathbf {M}$. We show now that by choosing $\mathbf {V}=V_0=0$ we arrive at the usual multipolar gauge Hamiltonian of the conventional theory. This means that choosing $\mathbf {V}=V_0=0$ must result in the same light/matter partition as that in the multipolar gauge of the conventional theory. After making this choice we can now remove the tildes on the fields. We must then also choose a gauge in order for the quantisation procedure to be well-defined. This is because there are redundant variables in the Lagrangian, just as in a free $\mathbf {A}$-field theory. Here we pick the Coulomb-gauge analogue of $\varvec{\nabla }\cdot \mathbf {C}=0$ and $C_0 = 0$, but we note that the gauge does not affect the light/matter partition nor the form of the Hamiltonian. We quantise the fields by enforcing $\left[ \widehat{C}^{\perp }_i(\mathbf {x}),\widehat{\Pi }_j(\mathbf {x}^{\prime })\right] =i\delta ^{\perp }_{ij}(\mathbf {x}-\mathbf {x}^{\prime })$, where

25 $$\begin{aligned} \widehat{\mathbf {C}}^{\perp }(\mathbf {x})=\sum _{\mathbf {k}\lambda }\varvec{\epsilon }_{\mathbf {k}\lambda }f_{\mathbf {k}}\left( \hat{\mathfrak {c}}_{\mathbf {k}\lambda }^{\dagger }{\mathrm {e}}^{-i\mathbf {k}\cdot \mathbf {x}}+\hat{\mathfrak {c}}_{\mathbf {k}\lambda }{\mathrm {e}}^{i\mathbf {k}\cdot \mathbf {x}}\right) , \end{aligned}$$

and $f_{\mathbf {k}}=(2\nu _{\mathbf {k}}V)^{-{1/2}}$ is the coupling strength to the mode with frequency $\nu _{\mathbf {k}}=c|\mathbf {k}|$ in volume *V*, $\varvec{\epsilon }_{\mathbf {k}\lambda }$ are polarisation vectors orthonormal to $\mathbf {k}$, and ${\hat{\mathfrak {c}}}_{\mathbf {k}\lambda }$ (${\hat{\mathfrak {c}}}_{\mathbf {k}\lambda }^{\dagger }$) is the photon annihilation (creation) operator for the $\mathbf {C}$-field. We note that for different choices of $\mathbf {V}$ and $V_0$, the ladder operators in $\widehat{{\mathbf {C}}}^{\perp }$ describe different bosons. After the matter parts of the Hamiltonian are also quantised and expanded into eigenstates (truncated to $N+1$ levels) the Hamiltonian can be written as

26 $$\begin{aligned} \widehat{H}= \sum _{n=0}^N \epsilon _{n}\left| \epsilon _{n}\right\rangle \left\langle \epsilon _{n}\right| +\sum _{\mathbf {k}\lambda }\nu _{\mathbf {k}} \hat{\mathfrak {c}}^{\dagger }_{\mathbf {k}\lambda }\hat{\mathfrak {c}}_{\mathbf {k}\lambda }-\frac{1}{\varepsilon _0}\widehat{\mathbf {d}}\cdot \left[ \varvec{\nabla }\times \widehat{\mathbf {C}}^{\perp }(\mathbf {0})\right] +\frac{1}{\varepsilon _0}\sum _{\mathbf {k}\lambda }f_{\mathbf {k}}^2\nu _{\mathbf {k}}\left( \widehat{\mathbf {d}}\cdot \varvec{\epsilon _{\mathbf {k}\lambda }}\right) ^2, \end{aligned}$$

where $\widehat{\mathbf {d}}=-e\hat{\mathbf {r}}$. To arrive at Eq. (26), we make the electric dipole approximation (EDA) ($\mathbf {k}\cdot \mathbf {r}\ll 1$) which allows us to evaluate the fields at the origin, set $\mathbf {P}\simeq -e\mathbf {r}\delta (\mathbf {x})$ and ignore the smaller magnetisation interactions governed through $\varvec{\Phi }_0$. The quantization process is analogous to the $\mathbf {A}$-field theory, which is given in detail in Supplementary Material 3. We are now free to choose polarisation vectors in such a way that the polarisation of the physical fields as computed using $\mathbf {C}$ and $\mathbf {A}$-fields overlap. It then follows that the $\mathbf {C}$-field Hamiltonian in Eq. (26) has the same mathematical form as the multipolar gauge Hamiltonian for the $\mathbf {A}$-field, which is a reflection of the light/matter partitions being identical. In Table 1 we highlight the differences between the $\mathbf {A}$- and $\mathbf {C}$-field approaches.

**Table 1:** Comparison of $\mathbf {A}$-field and $\mathbf {C}$-field representations.

$\mathbf {A}$-field approach	$\mathbf {C}$-field approach
$\mathbf {B}=\varvec{\nabla }\times \mathbf {A}$	$\mathbf {H}=\varvec{\nabla }C_0+\dot{\mathbf {C}}$
$\mathbf {E}=-\varvec{\nabla }A_0-\dot{\mathbf {A}}$	$\mathbf {D}=\varvec{\nabla }\times \mathbf {C}$
$$\mathbf {A}^{\prime }= {\left\{ \begin{array}{ll} \mathbf {A}^{\perp }& C \\ \varvec{\Phi}_0 /e & m \end{array}\right. }$$	$$\mathbf {C}^{\prime }= {\left\{ \begin{array}{ll} \mathbf {C}^{\perp }& ``C'' \\ \varvec{\Phi }_{\mathbf {D}}/e & ``m'' \end{array}\right. }$$
$$\varvec{\Pi }^{\prime }= {\left\{ \begin{array}{ll} -\varepsilon _0\mathbf {E}^{\perp }& C \\ -\mathbf {D} & m \end{array}\right. }$$	$\widetilde{\varvec{\Pi }}= \mathbf {B}$
$$\mathbf {p}^{\prime }={\left\{ \begin{array}{ll} m\dot{\mathbf {r}}+e\mathbf {A}^{\perp }& C \\ m\dot{\mathbf {r}}-\varvec{\Phi }_0 & m \end{array}\right. }$$	$\widetilde{\mathbf {p}}=m\dot{\mathbf {r}}-\varvec{\Phi }_0-\widetilde{\varvec{\Phi }}$

Gauge dependent parameters are denoted with a prime. *C*/*m* (“*C*/*m*”) denotes the Coulomb/multipolar gauge (-analogues) in the $\mathbf {A}$-field ($\mathbf {C}$-field) representation respectively, however the choice of gauge is inconsequential for predictions of the $\mathbf {C}$-theory. We define $\varvec{\Phi }_{\mathbf {D}}(\mathbf {r})=\int d^3x\ \varvec{\theta }(\mathbf {x},\mathbf {r})\times \mathbf {D}(\mathbf {x})$.

## Accuracy of two-level truncations

**Figure 2:**
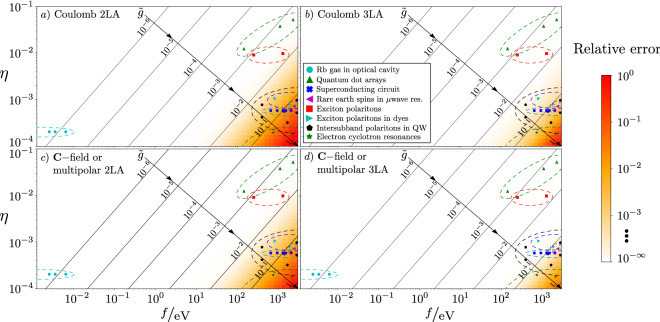
The relative error in calculating the lowest energy spacing of the full Coulomb gauge and $\mathbf {C}$-field Hamiltonians for an infinite square well potential. The axes are $\eta =(1/2\pi )x_{10}\nu = x_{10}/\lambda _{\mathrm {rad}}$, where $x_{{10}} = \left\langle {\epsilon _{0}^{\prime } \left| {\mathbf{x}} \right|\epsilon _{1}^{\prime } } \right\rangle$ is the approximate dipole size. The electric dipole approximation is satisfied when $\eta \ll 1$. To vary $\eta$, we vary $\nu$ whereas $x_{10}$ is constrained such that the first dipole transition is resonant with the radiation mode ($\epsilon _1-\epsilon _0=\nu$), which in practice results in tuning the length of the well. Along the horizontal axis, we plot the magnitude of the vector potential *f*. Importantly, the physically relevant second axis $\tilde{g} = g^C_{10}/\omega _{10}=g^m_{10}/\nu$ is plotted on the diagonal where $g^i_{10}$ is the transition strength between the two lowest lying states in gauge *i*. Here $\tilde{g}>1$ indicates ultra-strong coupling. We also show approximate regions where different types of QED experiments sit with respect to $\eta$ and $\tilde{g}$ in the plot, with markers indicating individual experiments given in Table SM1 of Supplementary Material 5. References for the experimental data: *Rb gas in optical cavity*^35–38^; *Quantum dot arrays*^39–43^; *Superconducting circuits*^44–48^; *Rare earth spins in* $\mu$*wave resonator*^49,50^; *Exciton polaritons*^49,50^; *Exciton polaritons in dyes*^51–54^; *Intersubband polaritons in quantum wells*^55–59^; *Electron cyclotron resonances*^60–63^.

We now turn to the question of whether a 2LT for the matter system is possible. In Fig. 2 we display the accuracy of the $\mathbf {C}$-field Hamiltonian in an arbitrary gauge, with $\mathbf {P}^\perp$ and $\mathbf {M}$ chosen such that the light/matter partition is equivalent to the $\mathbf {A}$-field in the multipolar gauge, along with the conventional $\mathbf {A}$-field in the Coulomb gauge. In both cases we truncate to two or three dipole levels, which we give details on shortly. Here, we use only a single radiation mode that is resonant with the transition between the two lowest dipole levels, and $\widehat{U}_{\mathrm {ext}}$ is an infinite square well potential whose anharmonicity makes it amenable to few-level expansion. In the strong coupling limit, $\tilde{g}\rightarrow 1$, both truncated Hamiltonians become inaccurate, importantly for different reasons. As discussed in Ref. ^9^, we expect a theory that limits the coupling between states far separated in energy space to most accurately model the physics, such as our $\mathbf {C}$-field Hamiltonian with $(\mathbf {V},V_0)=(\mathbf {0},0)$ or, equivalently, a multipolar $\mathbf {A}$-field Hamiltonian. In such a theory, we expect the dynamics to be limited to a manifold containing few states, and so accuracy is much improved by going from two to three levels. In contrast, the Coulomb gauge couples many energy states, and should thus be inaccurate when truncated to two, or three, levels.

We here appeal to discussion of the physics of the situation as the most natural way of determining a sensible light/matter partition. Importantly, there are two length-scales of the problem: the size of the dipole *L* and the wavelength of the light $\lambda$. First, for polarisation fields to be well-approximated by a dipole moment at the origin, i.e. $\mathbf {P}\propto \widehat{\mathbf {d}}\delta (\mathbf {x})$, we must have $\lambda \gg L$. Polarisation fields can, of course, be used nonetheless, but at a computational cost. Second, it is easy to see that the transition dipole moment $\mathbf {d}_{n,m}$ scales with the size of the dipole *L*, as $|\langle \widehat{\mathbf {d}}\rangle | \propto \left| \left\langle \hat{\mathbf {r}}\right\rangle \right| \propto L$. Similarly, the momentum expectation value must scale as 1/*L*, from unit considerations. Thus for a small dipole where $L \ll \lambda$, the momentum matrix elements $\mathbf {p}_{n,m}$ become large, whereas the dipole matrix elements $\mathbf {d}_{n,m}$ are small. This therefore necessarily limits the coupling between well-separated energy states when relying on a dipolar coupling, allowing the dynamics to take place in a small energy manifold. For a large dipole, the situation is reversed, and we should note that the polarisation field becomes computationally more intensive to use in the same limit (i.e. higher order multipolar modes must be accounted for). This suggests a physical origin to the success of the $\mathbf {C}$-field/multipolar gauge $\mathbf {A}$-field Hamiltonians. Indeed, we only see limited improvement by going from two to three levels for the Coulomb gauge. This is further discussed in Supplementary Material 6 where we repeat the calculation with a non-resonant cavity mode, shown in Fig. SM1. We should finally also note that in both cases we must keep counter-rotating terms, as they contribute significantly in the strong-coupling regime^65^.

We now give details on the numerics performed in Fig. 2. The Hamiltonians have Hilbert spaces $\mathcal {H}_m\otimes \mathcal {H}_p$, where $\mathcal {H}_m$ ($\mathcal {H}_p$) is the $N_m$ ($N_p$) dimensional Hilbert space of the matter (single mode photon field). Written in matrix form the Coulomb gauge Hamiltonian of the conventional QED formulation is

27 $$\begin{aligned} \varvec{\mathcal {H}}_{\mathrm {Cb}}=\varvec{\mathcal {H}}_m\otimes \mathbf {I}_{N_p}+\frac{e }{m}\mathbf {p}\otimes \mathbf {A}+\frac{e^2}{2m}\mathbf {I}_{N_m}\otimes \mathbf {A}^2+\nu \mathbf {I}_{N_m}\otimes \mathbf {a}^{\dagger }\cdot \mathbf {a} , \end{aligned}$$

where *e* and *m* are the electron charge and mass, $\nu$ is the energy of the photon mode and $\mathbf {I}_{d}$ is the identity operator of dimension *d*. Equation (27) is derived in Supplementary Material 1. The vector potential is $\mathbf {A}=f(\mathbf {a}+\mathbf {a}^{\dagger })$ where $\mathbf {a}$ is the annihilation operator matrix of dimension $N_p$ and *f* is the field amplitude. Note that throughout this example we assume that the dipole aligns with the polarisation of the field mode. The matter energy levels are contained within

28 $$\begin{aligned} \varvec{\mathcal {H}}_m=\sum _{n=1}^{N_m}\epsilon _{n}\left| \epsilon _{n}\right\rangle \left\langle \epsilon _{n}\right| , \end{aligned}$$

where $|\epsilon _n\rangle$ and $\epsilon _n$ are the eigenstate and eigenenergy solutions to the Schrödinger equation, and *n* is an integer. In Fig. 2 we use the one dimensional infinite square well potential which is zero within $0\le x\le L$ and infinite outside this range. This leads to the well known eigenenergies and position space wavefunctions

29 $$\begin{aligned}&\epsilon _n=\frac{\pi ^2 n^2}{2mL^2}, \end{aligned}$$

30 $$\psi _{n} (x) = \langle x|\epsilon _{n} \rangle = \sqrt {\frac{2}{L}} \sin \left( {\frac{{n\pi x}}{L}} \right).$$

Finally, the momentum matrix is $\mathbf {p}=\sum _{n,m=1}^{N_m}p_{n,m}\left| \epsilon _n\right\rangle \left\langle \epsilon _m\right|$ where the matrix elements are

31 $$\begin{aligned} p_{n,m}={\left\{ \begin{array}{ll} \frac{4\hbar }{iL}\frac{nm}{n^2-m^2} & n+m\text { odd} \\ 0 & n+m\text { even}, \end{array}\right. } \end{aligned}$$

with *n*, *m* integer. For a detailed reference on the infinite square well see Ref. ^66^.

For a single photon mode the $\mathbf {C}$-field Hamiltonian with $(\mathbf {V},V_0)=(\mathbf {0},0)$ (and equivalently multipolar of the conventional QED formulation) is

32 $$\begin{aligned} \varvec{\mathcal {H}}_{\mathbf {C}-\mathrm {field}}= \varvec{\mathcal {H}}_m\otimes \mathbf {I}_{N_p}-\frac{1}{\varepsilon _0}\mathbf {d}\otimes \mathbf {D} +\frac{1}{\varepsilon _0}f^2\nu \mathbf {d}^2\otimes \mathbf {I}_{N_p}+\nu \mathbf {I}_{N_m}\otimes \varvec{\mathfrak {c}}^{\dagger }\cdot \varvec{\mathfrak {c}}, \end{aligned}$$

where $\mathbf {D}=\varvec{\nabla }\times \mathbf {C}=-if(\varvec{\mathfrak {c}}^{\dagger }-\varvec{\mathfrak {c}})$, $\varvec{\mathfrak {c}}$ is the $N_{\mathrm {p}}$-dimensional $\mathbf {C}$-field photon annihilation matrix and the dipole matrix $\mathbf {d}=-e\mathbf {x}$ where $\mathbf {x}=\sum _{n,m=1}^{N_m}x_{n,m}\left| \epsilon _n\right\rangle \left\langle \epsilon _m\right|$ with elements

33 $$\begin{aligned} x_{n,m}={\left\{ \begin{array}{ll} -\frac{8L}{\pi ^2}\frac{nm}{(n^2-m^2)^2} & n+m\text { odd} \\ 0 & n+m\text { even } (n\ne m)\\ L/2 & n=m. \end{array}\right. } \end{aligned}$$

In Fig. 2 we compare the error in truncating the matter Hilbert spaces to $N_m=2$, which is the 2LT, for the Coulomb and $\mathbf {C}$-field $(\mathbf {V},V_0)=(\mathbf {0},0)$ Hamiltonians. To do so we calculate the energy difference of the two lowest eigenstates in the matrices of Eqs. (27) and (32), working in units $\hbar =1=\varepsilon _0$. For both Hamiltonians we do this for $N_m=2$ and $N_m$ large enough for convergence of this energy transition. The latter is the same for both gauges and gives the exact gauge-independent value for this energy transition. For all calculations $N_p$ is also large enough such that the transition is converged with respect to this.

## Discussion

We stress that we are free to work in any $\mathbf {C}$-field gauge without affecting the light/matter partitioning, which may offer additional freedom. The $\mathbf {C}$-field gauge should also be chosen to reflect the physical situation: for instance if the system centre-of-mass is moving a Lorenz gauge is appropriate, whereas a Coulomb gauge is a good choice for static systems. The latter may be useful also if boundaries between different regions are considered, which is not the case for $\mathbf {A}$-field Coulomb gauge where a generalisation is required to make the problem tractable^67^. In the example of a small dipole in a cavity, the $\mathbf {C}$-field aligns with the multipolar gauge in the $\mathbf {A}$-field representation, and so we agree with the conclusion of Refs.^9,10,14^ that this $\mathbf {A}$-field gauge choice most accurately represents the physics of small, bound dipoles.

The equivalence between the $\mathbf {C}$-field and $\mathbf {A}$-field approaches warrants further consideration. For example, what choice of $\mathbf {V}$ and $V_0$ leads to a $\mathbf {C}$-field Hamiltonian that is analogous to the Coulomb gauge of the $\mathbf {A}$-field approach? Additionally, it would be interesting to find the set of $(\mathbf {V},V_0)$ transformations that are allowed, i.e. that cause Eq. (24) to vanish.

In conclusion, we find that for systems without free currents and where a truncation of the matter system to few levels is desirable – typical of cavity QED situations – the $\mathbf {C}$-field representation is an attractive choice: it completely removes the dependence of physical predictions after a level truncation on the choice of gauge. In other words, in the $\mathbf {C}$-field representation, a gauge transformation does not set the light/matter partition. Instead this freedom is moved into the choice of $\mathbf {P}^{\perp }$ and $\mathbf {M}$, which may be a more attractive choice. The $\mathbf {C}$-field approach to QED offers an alternative route, distinct from gauge-fixing in the conventional $\mathbf {A}$-field representation, to choosing the correct light/matter partition for a given system. In any case, the accuracy of results obtained in the (matter-truncated) $\mathbf {C}$-representation are independent of the choice of gauge and limited only by the validity of the few-level truncation, decided by the chosen light/matter partition and the system being modelled.

## Supplementary information

Supplementary Infromation

## References

[1] Fiutak J (1963). The multipole expansion in quantum theory. Can. J. Phys..

[2] Babiker M, Loudon R (1983). Derivation of the Power–Zienau–Woolley Hamiltonian in quantum electrodynamics by gauge transformation. Proc. R. Soc. Lond. A Math. Phys. Sci..

[3] Jackson JD (2002). From Lorenz to Coulomb and other explicit gauge transformations. Am. J. Phys..

[4] Kok P, Lovett BW (2010). Introduction to Optical Quantum Information Processing.

[5] Mahan GD (2013). Many-Particle Physics.

[6] Rousseau E, Felbacq D (2017). The quantum-optics Hamiltonian in the Multipolar gauge. Sci. Rep..

[7] Andrews DL, Jones GA, Salam A, Woolley RG (2018). Perspective: Quantum Hamiltonians for optical interactions. J. Chem. Phys..

[8] Stokes A, Nazir A (2019). Gauge ambiguities imply Jaynes–Cummings physics remains valid in ultrastrong coupling QED. Nat. Commun..

[9] De Bernardis D, Pilar P, Jaako T, De Liberato S, Rabl P (2018). Breakdown of gauge invariance in ultrastrong-coupling cavity QED. Phys. Rev. A.

[10] De Bernardis D, Jaako T, Rabl P (2018). Cavity quantum electrodynamics in the nonperturbative regime. Phys. Rev. A.

[11] Stokes A, Nazir A (2018). A master equation for strongly interacting dipoles. New J. Phys..

[12] Vukics, A., Kónya, G. & Domokos, P. The gauge-invariant Lagrangian, the Power-Zienau–Woolley picture, and the choices of field momenta in nonrelativistic quantum electrodynamics. arXiv preprint arXiv:1801.05590 (2018). 10.1038/s41598-021-94405-z PMC8358002 34381077

[13] Rousseau, E., & Felbacq, D. Reply to “The gauge-invariant Lagrangian, the Power–Zienau–Woolley picture, and the choices of field momenta in nonrelativistic quantum electrodynamics.” by A. Vuckis et al. https://hal.archives-ouvertes.fr/hal-01760460/ (2019). 10.1038/s41598-021-94405-z PMC8358002 34381077

[14] Di Stefano O (2019). Resolution of gauge ambiguities in ultrastrong-coupling cavity quantum electrodynamics. Nat. Phys..

[15] Göppert-Mayer M (1931). Über elementarakte mit zwei quantensprüngen. Ann. Phys..

[16] Power EA, Zienau S, Massey HSW (1959). Coulomb gauge in non-relativistic quantum electro-dynamics and the shape of spectral lines. Philos. Trans. R. Soc. Lond. Ser. A Math. Phys. Sci..

[17] Yang K-H (1976). Gauge transformations and quantum mechanics I. Gauge invariant interpretation of quantum mechanics. Ann. Phys..

[18] Ackerhalt JR, Milonni PW (1984). Interaction Hamiltonian of quantum optics. JOSA B.

[19] Lamb WE, Schlicher RR, Scully MO (1987). Matter-field interaction in atomic physics and quantum optics. Phys. Rev. A.

[20] Cohen-Tannoudji C, Dupont-Roc J, Grynberg G (1997). Photons and Atoms-Introduction to Quantum Electrodynamics.

[21] Rokaj V, Welakuh DM, Ruggenthaler M, Rubio A (2018). Light-matter interaction in the long-wavelength limit: No ground-state without dipole self-energy. J. Phys. B At. Mol. Opt. Phys..

[22] Schäfer C, Ruggenthaler M, Rokaj V, Rubio A (2020). Relevance of the quadratic diamagnetic and self-polarization terms in cavity quantum electrodynamics. ACS Photon..

[23] Settineri, A., Stefano, O. D., Zueco, D., Hughes, S., Savasta, S., & Nori, F. Gauge freedom, quantum measurements and time-dependent interactions in cavity and circuit QED. arXiv preprint arXiv:1912.08548 (2019).

[24] Garziano, L., Settineri, A., Di Stefano, O., Savasta, S., & Nori, F. Gauge invariance of the Dicke and Hopfield models. arXiv preprint arXiv:2002.04241 (2020).

[25] Taylor, A., Manda, A., Zhou, W., & Huo, P. Gauge Invariance in Molecular Cavity Quantum Electrodynamics. arXiv preprint arXiv:2006.03191 (2020).

[26] Roth M, Hassler F, DiVincenzo DP (2019). Optimal gauge for the multimode Rabi model in circuit QED. Phys. Rev. Res..

[27] Stokes, A., & Nazir, A. Ultrastrong time-dependent light-matter interactions are gauge-relative. arXiv preprint arXiv:1902.05160 (2019b).

[28] Bliokh KY, Bekshaev AY, Nori F (2013). Dual electromagnetism: Helicity, spin, momentum and angular momentum. New J. Phys..

[29] Baker, M., Ball, J. S., & Zachariasen, F. Classical electrodynamics with dual potentials. arXiv preprint arXiv:hep-th/9403169 (1994).

[30] Bisht P, Negi O (2008). Revisiting quaternion dual electrodynamics. Int. J. Theor. Phys..

[31] Chiao RY, Hansson TH, Leinaas JM, Viefers S (2004). Effective photon-photon interaction in a two-dimensional "photon fluid". Phys. Rev. A.

[32] Hillery M, Mlodinow LD (1984). Quantization of electrodynamics in nonlinear dielectric media. Phys. Rev. A.

[33] Drummond PD, Hillery M (2014). The Quantum Theory of Nonlinear Optics.

[34] Jackson, J. D. “Classical electrodynamics” (1999).

[35] Suleymanzade, A., Anferov, A., Stone, M., Naik, R. K., Simon, J., & Schuster, D. A tunable High-Q millimeter wave cavity for hybrid circuit and cavity QED experiments. arXiv preprint arXiv:1911.00553 (2019).

[36] Colombe Y (2007). Strong atom-field coupling for Bose–Einstein condensates in an optical cavity on a chip. Nature.

[37] Thompson JD (2013). Coupling a single trapped atom to a nanoscale optical cavity. Science.

[38] Tiecke T (2014). Nanophotonic quantum phase switch with a single atom. Nature.

[39] Gérard J (1998). Enhanced spontaneous emission by quantum boxes in a monolithic optical microcavity. Phys. Rev. Lett..

[40] Gerard J (1996). Quantum boxes as active probes for photonic microstructures: The pillar microcavity case. Appl. Phys. Lett..

[41] Gerard J (1998). InAs quantum boxes in GaAs/AlAs pillar microcavities: From spectroscopic investigations to spontaneous emission control. Phys. E Low-Dimens. Syst. Nanostruct..

[42] Gayral B (1999). High-Q wet-etched GaAs microdisks containing InAs quantum boxes. Appl. Phys. Lett..

[43] Moreau E (2001). Single-mode solid-state single photon source based on isolated quantum dots in pillar microcavities. Appl. Phys. Lett..

[44] Johansson J (2006). Vacuum Rabi oscillations in a macroscopic superconducting qubit LC oscillator system. Phys. Rev. Lett..

[45] Niemczyk T (2010). Circuit quantum electrodynamics in the ultrastrong-coupling regime. Nat. Phys..

[46] Forn-Díaz P (2010). Observation of the Bloch–Siegert shift in a qubit-oscillator system in the ultrastrong coupling regime. Phys. Rev. Lett..

[47] Baust A (2016). Ultrastrong coupling in two-resonator circuit QED. Phys. Rev. B.

[48] Yoshihara F (2017). Superconducting qubit-oscillator circuit beyond the ultrastrong-coupling regime. Nat. Phys..

[49] Weisbuch C, Nishioka M, Ishikawa A, Arakawa Y (1992). Observation of the coupled exciton-photon mode splitting in a semiconductor quantum microcavity. Phys. Rev. Lett..

[50] Bloch J, Freixanet T, Marzin J, Thierry-Mieg V, Planel R (1998). Giant Rabi splitting in a microcavity containing distributed quantum wells. Appl. Phys. Lett..

[51] Bellessa J, Bonnand C, Plenet J, Mugnier J (2004). Strong coupling between surface plasmons and excitons in an organic semiconductor. Phys. Rev. Lett..

[52] Wei H-S (2013). Adjustable exciton-photon coupling with giant Rabi-splitting using layer-by-layer J-aggregate thin films in all-metal mirror microcavities. Opt. Express.

[53] Gambino S (2014). Exploring light-matter interaction phenomena under ultrastrong coupling regime. ACS Photon..

[54] Kéna-Cohen S, Maier SA, Bradley DD (2013). Ultrastrongly coupled exciton-polaritons in metal-clad organic semiconductor microcavities. Adv. Opt. Mater..

[55] Dupont E, Liu H, SpringThorpe A, Lai W, Extavour M (2003). Vacuum-field Rabi splitting in quantum-well infrared photodetectors. Phys. Rev. B.

[56] Dupont E, Gupta J, Liu H (2007). Giant vacuum-field Rabi splitting of intersubband transitions in multiple quantum wells. Phys. Rev. B.

[57] Todorov Y (2010). Ultrastrong light-matter coupling regime with polariton dots. Phys. Rev. Lett..

[58] Delteil A (2012). Charge-induced coherence between intersubband plasmons in a quantum structure. Phys. Rev. Lett..

[59] Askenazi B (2014). Ultra-strong light-matter coupling for designer Reststrahlen band. New J. Phys..

[60] Muravev V, Andreev I, Kukushkin I, Schmult S, Dietsche W (2011). Observation of hybrid plasmon-photon modes in microwave transmission of coplanar microresonators. Phys. Rev. B.

[61] Scalari G (2012). Ultrastrong coupling of the cyclotron transition of a 2D electron gas to a THz metamaterial. Science.

[62] Maissen C (2014). Ultrastrong coupling in the near field of complementary split-ring resonators. Phys. Rev. B.

[63] Bayer A (2017). Terahertz light-matter interaction beyond unity coupling strength. Nano Lett..

[64] Drummond PD (2006). Dual-symmetric Lagrangians in quantum electrodynamics: I. Conservation laws and multi-polar coupling. J. Phys. B At. Mol. Opt. Phys..

[65] Feranchuk, I., San, N., Leonau, A., & Skoromnik, O. Exact solution for the quantum Rabi model with the $$\varvec {\sf A}^{2}$$ term. arXiv preprint arXiv:2002.03702 (2020).

[66] Prentis J, Ty B (2014). Matrix mechanics of the infinite square well and the equivalence proofs of Schrödinger and von Neumann. Am. J. Phys..

[67] Zietal R, Eberlein C (2019). Gauge transformation in macroscopic quantum electrodynamics near polarizable surfaces. Phys. Rev. D.

[68] Rouse, D. M., Lovett, B. W., Gauger, E. & Westerberg, N. Avoiding gauge ambiguities in cavity quantum electrodynamics (dataset). Dataset. University of St Andrews Research Portal. 10.17630/9b64c99d-b49b-455c-b439-c896f5cd0f2b (2021). 10.1038/s41598-021-83214-z PMC7896096 33608609

