# Supplementary material for: Avoiding gauge ambiguities in cavity quantum electrodynamics
Source: Sci Rep. 2021 Feb 19;11:4281. doi: 10.1038/s41598-021-83214-z (PMC7896096; doi:10.1038/s41598-021-83214-z)
Supplement: Supplementary file 1 — Supplementary Infromation [file 41598_2021_83214_MOESM1_ESM.pdf]

Avoiding gauge ambiguities in cavity quantum electrodynamics

Dominic M. Rouse,[1] Brendon W. Lovett,[1] Erik M. Gauger,[2] and Niclas Westerberg[3, 4]
[1] *SUPA, School of Physics and Astronomy, University of St Andrews, St Andrews, KY16 9SS, UK*
[2] *SUPA, Institute of Photonics and Quantum Sciences, Heriot-Watt University, Edinburgh, EH14 4AS, UK*
[3] *School of Physics and Astronomy, University of Glasgow, Glasgow G12 8QQ, United Kingdom*
[4] *SUPA, Institute of Photonics and Quantum Sciences, Heriot-Watt University, Edinburgh, EH14 4AS, UK*
**dmr9@st-andrews.ac.uk*
† *Niclas.Westerberg@glasgow.ac.uk*
(Dated: November 30, 2020)

SUPPLEMENTARY MATERIAL 1: THE COULOMB AND THE MULTIPOLAR GAUGES FOR THE SIMPLE DIPOLE SYSTEM

We start from the arbitrary-gauge Hamiltonian given in Eqn. (9) of the main text (repeated here for ease):

$$H' = \sum_\mu \frac{1}{2m_\mu}\left[\mathbf{p}'_\mu - q_\mu \mathbf{A}(\mathbf{r}_\mu) + q_\mu \boldsymbol{\nabla}\chi(\mathbf{r}_\mu)\right]^2 + \int d^3x \ \left(\frac{1}{2\varepsilon_0}\left[\boldsymbol{\Pi}'(\mathbf{x}) + \boldsymbol{\phi}'(\mathbf{x})\right]^2 + \frac{\mathbf{B}^2(\mathbf{x})}{2\mu_0}\right).$$

Despite superficial differences the theory is still gauge invariant and so, irrespective of the choice of χ-field, H'_m will describe the same physics. From Eqn. (5b) it is clear that the transverse part of the vector potential is gauge invariant. This is quantised in the usual way by satisfying the commutation relation $\left[\widehat{A}'^{\perp}_i(\mathbf{x}), \widehat{\Pi}'^{\perp}_j(\mathbf{x}')\right] = i\delta^{\perp}_{ij}(\mathbf{x}-\mathbf{x}')$, where $\delta^{\perp}_{ij}(\mathbf{x}-\mathbf{x}')$ is the transverse δ-function, yielding

$$\widehat{\mathbf{A}}^{\perp}(\mathbf{x}) = \sum_{\mathbf{k}\lambda} \boldsymbol{\epsilon}_{\mathbf{k}\lambda} f_{\mathbf{k}} \left(\hat{a}'^{\dagger}_{\mathbf{k}\lambda} \mathrm{e}^{-i\mathbf{k}\cdot\mathbf{x}} + \hat{a}'_{\mathbf{k}\lambda}\mathrm{e}^{i\mathbf{k}\cdot\mathbf{x}}\right), \tag{SM 1}$$

where $f_{\mathbf{k}} = (2\nu_{\mathbf{k}}V)^{-1/2}$ is the coupling strength to the mode with frequency $\nu_{\mathbf{k}} = c|\mathbf{k}|$ in volume V, $\boldsymbol{\epsilon}_{\mathbf{k}\lambda}$ are polarisation unit vectors orthonormal to $\mathbf{k}$, with $\hat{a}'_{\mathbf{k}\lambda}$ ($\hat{a}'^{\dagger}_{\mathbf{k}\lambda}$) being a gauge-dependent photon annihilation (creation) operator [1–4]. In the following we will henceforth derive equations for the simple dipole system in the main text instead of the generic charge distribution. From Eqn. (SM 1) the magnetic field follows as

$$\begin{aligned}\widehat{\mathbf{B}}(\mathbf{x}) &= \boldsymbol{\nabla}\times\widehat{\mathbf{A}}(\mathbf{x}) \\ &= -i\sum_{\mathbf{k}\lambda}\left(\mathbf{k}\times\boldsymbol{\epsilon}_{\mathbf{k}\lambda}\right) f_{\mathbf{k}}\left(\hat{a}'^{\dagger}_{\mathbf{k}\lambda}\mathrm{e}^{-i\mathbf{k}\cdot\mathbf{x}} - \hat{a}'_{\mathbf{k}\lambda}\mathrm{e}^{i\mathbf{k}\cdot\mathbf{x}}\right).\end{aligned} \tag{SM 2}$$

The canonical momentum of the field is gauge dependent, but has the same expression in each gauge found through the commutator $\left[\widehat{A}'^{\perp}_i(\mathbf{x}), \widehat{\Pi}'^{\perp}_j(\mathbf{x}')\right] = i\delta^{\perp}_{ij}(\mathbf{x}-\mathbf{x}')$, where $\delta^{\perp}_{ij}(\mathbf{x}-\mathbf{x}')$, and is given by

$$\widehat{\boldsymbol{\Pi}}'^{\perp}(\mathbf{x}) = i\sum_{\mathbf{k}\lambda}\nu_{\mathbf{k}}\boldsymbol{\epsilon}_{\mathbf{k}\lambda} f_{\mathbf{k}}\left(\hat{a}'^{\dagger}_{\mathbf{k}\lambda}\mathrm{e}^{-i\mathbf{k}\cdot\mathbf{x}} - \hat{a}'_{\mathbf{k}\lambda}\mathrm{e}^{i\mathbf{k}\cdot\mathbf{x}}\right). \tag{SM 3}$$

A. Coulomb gauge

The Coulomb gauge Hamiltonian is defined by choosing $\boldsymbol{\nabla}\chi = \mathbf{A}^{\parallel}$ and results in $\boldsymbol{\phi}_C = -\varepsilon_0\mathbf{E}^{\parallel}$ and so $\boldsymbol{\Pi}_C = -\varepsilon_0\mathbf{E}^{\perp}$ where we have replaced superscript 'prime' with subscript 'C' to denote the gauge [5]. The photon field is purely transverse with $\mathbf{A}_C = \mathbf{A}^{\perp}$ with conjugate field $\boldsymbol{\Pi}_C = -\varepsilon_0\mathbf{E}^{\perp}$. This also means that the Coulomb gauge canonical momentum is

$$\mathbf{p}_{C,\mu} = m_\mu\dot{\mathbf{r}}_\mu - q_\mu\mathbf{A}^{\perp}(\mathbf{r}_\mu). \tag{SM 4}$$

On substituting these into the arbitrary-gauge Hamiltonian Eqn. (SM 1) for the simple system described in the main text, one finds that

$$H_C = \frac{1}{2m}\left[\mathbf{p}_C + e\mathbf{A}^{\perp}(\mathbf{r})\right]^2 + U_{\text{ext}} + U_b + \frac{1}{2}\int d^3x\ \left(\frac{1}{\varepsilon_0}\left[\mathbf{\Pi}_C^{\perp}(\mathbf{x})\right]^2 + \frac{1}{\mu_0}\left[\mathbf{B}(\mathbf{x})\right]^2\right),$$

where U_{ext} is an externally applied potential and $U_b = \frac{\varepsilon_0}{2}\int d^3x\ [\mathbf{E}^{\parallel}(\mathbf{x})]^2$ is the electron-hole electrostatic potential which contains the divergent self-energies and interaction between the particles. This potential follows from Gauss's law Eqn. (1c) and the lack of free charge, which leads to

$$\begin{aligned}\mathbf{E}^{\parallel}(\mathbf{x}) &= -\boldsymbol{\nabla}\int d^3x'\ \frac{\rho_b(\mathbf{x}')}{4\pi\varepsilon_0\left|\mathbf{x}-\mathbf{x}'\right|} \\ &\equiv -\frac{1}{\varepsilon_0}\boldsymbol{\nabla}V_b(\mathbf{x}),\end{aligned} \tag{SM 5}$$

where $V_b = V_e + V_h$ is the sum of electron and hole potentials. Integration by parts, assuming fields vanish at infinities, and again using Gauss's law leads to

$$\begin{aligned}U_b &= \frac{1}{2\varepsilon_0}\int d^3x\ \rho_b(\mathbf{x})V_b(\mathbf{x}) \\ &\equiv U_{e-h} + U_{e-e} + U_{h-h},\end{aligned} \tag{SM 6}$$

where U_{e-e} and U_{h-h} are the divergent self energies of the electron and hole and U_{e-h} is the electrostatic energy due to the mutual attraction. The $\mathbf{A}^{\perp}$, $\mathbf{B}$ and $\mathbf{\Pi}_C^{\perp}$ fields are quantised as in Eqns. (SM 1), (SM 2) and (SM 3) albeit with the arbitrary gauge ladder operators replaced with Coulomb-gauge ones. Using these, the terms inside the integral in Eqn. (SM 5) becomes the usual bosonic field energy term and so the Coulomb-gauge Hamiltonian becomes

$$H_C = \frac{1}{2m}\left[\widehat{\mathbf{p}}_C + e\widehat{\mathbf{A}}^{\perp}(\mathbf{r})\right]^2 + \widehat{U}_{\text{ext}} + \widehat{U}_b + \sum_{\mathbf{k}\lambda}\nu_{\mathbf{k}}\left(a^{\dagger}_{\mathbf{k}\lambda}a_{\mathbf{k}\lambda} + \frac{1}{2}\right). \tag{SM 7}$$

In the Coulomb gauge, the light/matter coupling is between the dipole canonical momentum and the gauge invariant transverse vector potential, of the form $\widehat{\mathbf{p}}_C \cdot \widehat{\mathbf{A}}^{\perp}$. From Eqn. (7b), we see that the field canonical momentum is proportional to the transverse electric field, $\mathbf{\Pi}_C = -\varepsilon_0\mathbf{E}^{\perp}$. We now expand in the basis of $N+1$ energy eigenstates. The system Hamiltonian is formed from

$$\frac{\widehat{\mathbf{p}}_C^2}{2m} + \widehat{U}_{\text{ext}} \rightarrow \sum_{n=0}^{N}\epsilon_n\left|\epsilon_{n,C}\right\rangle\left\langle\epsilon_{n,C}\right| \equiv H_{0,C}. \tag{SM 8}$$

Finally, the Coulomb-gauge light matter interaction is written in this basis as follows

$$\begin{aligned}\frac{e}{m}\widehat{\mathbf{p}}\cdot\widehat{\mathbf{A}}^{\perp} &= ie\left[H_{0,C},\widehat{\mathbf{r}}\right]\cdot\widehat{\mathbf{A}}^{\perp} \\ &= -\sum_{n=0}^{N}\sum_{n'>n}^{N-1}\epsilon_{nn'}\mathbf{d}_{nn'}\cdot\widehat{\mathbf{A}}^{\perp}\widehat{\sigma}^{y}_{nn'},\end{aligned} \tag{SM 9}$$

where $\epsilon_{nn'} \equiv \epsilon_{n'} - \epsilon_n$, $\mathbf{d}_{nn'} = -e\left\langle\epsilon_{n,C}\right|\widehat{\mathbf{r}}\left|\epsilon_{n',C}\right\rangle$ and $\widehat{\sigma}^{y}_{nn'} = -i\left(\left|\epsilon_{n',C}\right\rangle\left\langle\epsilon_{n,C}\right| - \left|\epsilon_{n,C}\right\rangle\left\langle\epsilon_{n',C}\right|\right)$. In the second line of Eqn. (SM 9) we have written $\mathbf{p} = m\dot{\mathbf{r}}$, which is only true in Coulomb gauge in the absence of a vector potential, and is why the unperturbed Hamiltonian is used in the expectation value $\dot{\mathbf{r}} = i[H_0,\mathbf{r}]$. Finally, the Coulomb-gauge Hamiltonian written in the energy eigenbasis is

$$H_C = \sum_{n=0}^{N}\epsilon_n\left|\epsilon_{n,C}\right\rangle\left\langle\epsilon_{n,C}\right| - \sum_{n=0}^{N}\sum_{n'>n}^{N-1}\epsilon_{nn'}\mathbf{d}_{nn'}\cdot\widehat{\mathbf{A}}^{\perp}\widehat{\sigma}^{y}_{nn'} + \frac{1}{2m}\left[e\widehat{\mathbf{A}}^{\perp}\right]^2 + \sum_{\mathbf{k}\lambda}\nu_{\mathbf{k}}\hat{a}^{\dagger}_{\mathbf{k}\lambda}\hat{a}_{\mathbf{k}\lambda}, \tag{SM 10}$$

where the ladder operators are implicitly in Coulomb gauge. We have made the electric dipole approximation which allows us to evaluate the vector potential at the centre of the dipole at the origin at the coordinate system, i.e. $\mathbf{A}^{\perp} \equiv \mathbf{A}^{\perp}(0)$. Additionally, we have ignored the vacuum energy of the photon field, and the divergent self energies of the electron and hole, $\widehat{U}_{e-e}$ and $\widehat{U}_{h-h}$, and also the electron-hole interaction $\widehat{U}_{e-h}$ which is itself divergent in the electric dipole approximation.

B. Multipolar gauge

The derivation of the multipolar gauge Hamiltonian is more involved and the reader is pointed to [5, 6] for the details. It can be summarised by the choice $\boldsymbol{\nabla}\chi = \mathbf{A}^{\perp} - \boldsymbol{\Phi}_0/e$. This leads to $\boldsymbol{\phi}_m = \mathbf{P}$ and therefore $\boldsymbol{\Pi}_m = -\mathbf{D} = -\mathbf{D}^{\perp}$ where the lack of longitudinal component follows from Gauss' law because there are no free charges. Subscript 'm' labels quantities in the multipolar gauge. By substitution into Eqn. (9), one finds that the Hamiltonian in the multipolar gauge is

$$H_m = \frac{1}{2m}\left[\mathbf{p}_m + \boldsymbol{\Phi}(\mathbf{r})\right]^2 + U_{\text{ext}} + \frac{1}{2}\int d^3x \left(\frac{1}{\varepsilon_0}\left[\boldsymbol{\Pi}_m^{\perp}(\mathbf{x}) + \mathbf{P}(\mathbf{x},\mathbf{r})\right]^2 + \frac{1}{\mu_0}\mathbf{B}^2(\mathbf{x})\right). \tag{SM 11}$$

The EM fields are quantised in the same way as in Coulomb-gauge, despite $\boldsymbol{\Pi}_m^{\perp}$ and $\boldsymbol{\Pi}_C^{\perp}$ corresponding to different physical fields. In the multipolar gauge, the electrostatic interaction is governed by the longitudinal component of the polarisation field $\widehat{U}_b = \frac{1}{2\varepsilon_0}\int d^3x\, [\widehat{\mathbf{P}}^{\parallel}(\mathbf{x})]^2$, which is clear from Eqn. (12a) and leads to the same expression given in Eqn. (SM 6). In the multipolar gauge, the photon field is much more complicated, $\boldsymbol{\Pi}_m = -\mathbf{D}^{\perp} = -\mathbf{E}^{\perp} - \mathbf{P}^{\perp}$, and contains both dipole and transverse field degrees of freedom. The light/matter coupling is between the mechanical dipole position and the canonical momentum of the field, $\int d^3x\, \widehat{\mathbf{P}}^{\perp}(\mathbf{x}) \cdot \widehat{\mathbf{D}}^{\perp}(\mathbf{x})$. Now expanding the matter into the energy basis, the interaction becomes

$$\begin{aligned}\int d^3x\, \widehat{\mathbf{P}}^{\perp}(\mathbf{x},\mathbf{r}) \cdot \widehat{\boldsymbol{\Pi}}_m^{\perp}(\mathbf{x}) &= -e\int d^3x \int_0^1 d\lambda\, \hat{\mathbf{r}} \cdot \widehat{\boldsymbol{\Pi}}_m^{\perp}(\mathbf{x})\delta(\mathbf{x}-\lambda\mathbf{r}) \\ &= -e\hat{\mathbf{r}} \cdot \int_0^1 d\lambda\, \widehat{\boldsymbol{\Pi}}_m^{\perp}(\lambda\mathbf{r}) \\ &\approx \widehat{\mathbf{d}} \cdot \widehat{\boldsymbol{\Pi}}_m^{\perp}(0),\end{aligned} \tag{SM 12}$$

where we used the dipole operator $\widehat{\mathbf{d}} = -e\hat{\mathbf{r}}$ and in the last line we have made the electric dipole approximation. Within the EDA, the dipolar self-energy term can be rewritten using the transverse delta function as [4]

$$\frac{1}{2}\int d^3x \left[\widehat{\mathbf{P}}^{\perp}(\mathbf{x},\mathbf{r})\right]^2 \approx \sum_{\mathbf{k}} f_{\mathbf{k}}^2 \nu_{\mathbf{k}} \left(\widehat{\mathbf{d}} \cdot \boldsymbol{\epsilon}_{\mathbf{k}\boldsymbol{\lambda}}\right)^2. \tag{SM 13}$$

Applying these, expanding the kinetic energy terms of the eigenbasis as in Eqn. (SM 8) and ignoring the magnetic contribution in $\boldsymbol{\Phi}$, we find the standard multipolar Hamiltonian

$$H_m = \sum_{n=0}^{N} \epsilon_n \left|\epsilon_{n,m}\right\rangle \left\langle\epsilon_{n,m}\right| - \frac{1}{\varepsilon_0}\widehat{\mathbf{d}} \cdot \widehat{\mathbf{D}}^{\perp}(0) + \frac{1}{\varepsilon_0}\sum f_{\mathbf{k}}^2 \nu_{\mathbf{k}} \left(\widehat{\mathbf{d}} \cdot \boldsymbol{\epsilon}_{\mathbf{k}\boldsymbol{\lambda}}\right)^2 + \sum_{\mathbf{k}\lambda} \nu_{\mathbf{k}} \hat{a}_{\mathbf{k}\lambda}^{\dagger} \hat{a}_{\mathbf{k}\lambda}, \tag{SM 14}$$

where the ladder operators are implicitly in multipolar gauge. The similarities between the multipolar Hamiltonian derived here in the **A**-field approach and the gauge-invariant Hamiltonian we have derived in the new **C**-field approach in Eqn. (26) should be noted (discussed further in the main text). Furthermore, the expansion of the dipole operator into the $N+1$ energy eigenstates is

$$\widehat{\mathbf{d}} = \sum_{n=0}^{N} \sum_{n'>n}^{N-1} \mathbf{d}_{nn'} \hat{\sigma}_{nn'}^{x}, \tag{SM 15}$$

where we have assumed that the binding potential gives rise to eigenstates for which the diagonal elements of the dipole transition matrix are zero and $\mathbf{d}_{nn'} = -e\left\langle\epsilon_{n,m}\right| \mathbf{r} \left|\epsilon_{n',m}\right\rangle$. Note that the coupling strength between levels n and n' scales like $d_{nn'}\nu$ in the multipolar gauge and $d_{nn'}\epsilon_{nn'}$ in Coulomb gauge. The increasing coupling strength with energy level separation in Coulomb gauge is related to the breakdown of gauge invariance for finite dipole level truncation, as reported by Refs. [7, 8].

SUPPLEMENTARY MATERIAL 2: ADDING AN IONIC LATTICE IN THE NEW APPROACH

An ionic lattice can be added to the new approach consistently under the assumption that it does not contribute any macroscopic current. This is justified given that ions vibrate about a mean position. To do so, we note that the

partitioning of ρ and $\mathbf{J}$ into free and bound charges was arbitrary. We could just as well make the partition $\rho = \rho_d + \rho_i$ and $\mathbf{J} = \mathbf{J}_d + \mathbf{J}_i$ where subscripts 'd' and 'i' denote charges that are bound in dipoles, and charges that are ions, respectively. The lattice is described by ions of charges Q_k at positions $\mathbf{R}_k$ with charge density

$$\rho_i\left(\mathbf{x}, \mathbf{R}_k\right) = \sum_k Q_k \delta(\mathbf{x} - \mathbf{R}_k), \tag{SM 16}$$

and $\mathbf{J}_i = 0$. Note that to conserve charge without a current density, the charge density must not be an explicit function of time.

A field $\boldsymbol{\mathcal{P}}$, analogous to the polarisation field $\mathbf{P}$, is then defined to be sourced by the dipoles: $\boldsymbol{\nabla} \cdot \boldsymbol{\mathcal{P}} = -\rho_d$ and a field $\boldsymbol{\mathcal{D}}$ analogous to the displacement field $\mathbf{D}$ to be sourced by the ions: $\boldsymbol{\nabla} \cdot \boldsymbol{\mathcal{D}} = \rho_i$. If there are no ions, then the new $\mathbf{C}$-field theory outlined in the main text is unchanged so long as $\mathbf{P} \to \boldsymbol{\mathcal{P}}$ and $\mathbf{D} \to \boldsymbol{\mathcal{D}}$ because $\boldsymbol{\nabla} \cdot \mathbf{E} = \rho$ is still satisfied for $\mathbf{E} = (\boldsymbol{\mathcal{D}} - \boldsymbol{\mathcal{P}})/\varepsilon_0$. Including the ions, the Maxwell equations Eqns. (13) become

$$\boldsymbol{\nabla} \cdot \boldsymbol{\mathcal{D}}^{\parallel}\left(\mathbf{x}, \mathbf{R}_k\right) = \rho_i\left(\mathbf{x}, \mathbf{R}_k\right), \tag{SM 17a}$$

$$\boldsymbol{\nabla} \times \mathbf{H}(\mathbf{x}) = \dot{\boldsymbol{\mathcal{D}}}^{\perp}(\mathbf{x}), \tag{SM 17b}$$

where we have set $\mathbf{J}_i = 0$. Analogously to the main text, we write the fields in terms of vector potentials to satisfy Eqns. (SM 17). As before $\mathbf{H} = \boldsymbol{\nabla} C_0 + \dot{\mathbf{C}}$, however, the displacement field analogue requires a longitudinal component

$$\boldsymbol{\mathcal{D}}\left(\mathbf{x}, \mathbf{R}_k\right) = \boldsymbol{\nabla} \times \mathbf{C}(\mathbf{x}) + \boldsymbol{\mathcal{D}}^{\parallel}\left(\mathbf{x}, \mathbf{R}_k\right), \tag{SM 18}$$

with the restrictions that $\boldsymbol{\nabla} \cdot \boldsymbol{\mathcal{D}}^{\parallel} = \rho_i$ and $\dot{\boldsymbol{\mathcal{D}}}^{\parallel} = 0$. The latter restriction comes from Eqn. (SM 17a) and the lack of a macroscopic free current. Note that as before $\boldsymbol{\mathcal{D}}^{\perp}(\mathbf{x}) = \boldsymbol{\nabla} \times \mathbf{C}(\mathbf{x})$. These restrictions are satisfied by choosing

$$\begin{aligned} \boldsymbol{\mathcal{D}}^{\parallel}\left(\mathbf{x}, \mathbf{R}_k\right) &= -\boldsymbol{\nabla} \int d^3x' \, \frac{\rho_i\left(\mathbf{x}', \mathbf{R}_k\right)}{4\pi \left|\mathbf{x} - \mathbf{x}'\right|} \\ &\equiv -\boldsymbol{\nabla} V_i\left(\mathbf{x}, \mathbf{R}_k\right), \end{aligned} \tag{SM 19}$$

where V_i is the electrostatic potential of the ionic lattice. The inclusion of an ionic lattice to the Lagrangian Eqn. (**??**) brings an additional kinetic term

$$L \to \bar{L} = L + \sum_k \frac{1}{2} M_k \dot{\mathbf{R}}_k^2, \tag{SM 20}$$

along with the replacements $\mathbf{P} \to \boldsymbol{\mathcal{P}}$ and $\mathbf{D}^{\perp} \to \boldsymbol{\mathcal{D}}^{\perp} + \boldsymbol{\mathcal{D}}^{\parallel}$ where $\boldsymbol{\mathcal{D}}^{\perp} = \mathbf{D}^{\perp} = \boldsymbol{\nabla} \times \mathbf{C}$. In order for the lattice to have been added consistently we must be able to derive the Lorentz force acting on the ions by using the Euler-Lagrange equation with ion position $\mathbf{R}_k$. The velocity derivative clearly results in $\partial \bar{L}/\partial \dot{\mathbf{R}}_k = M_k \dot{\mathbf{R}}_k$, though the position derivative is more difficult. To start we rewrite Eqn. (SM 19) as

$$\begin{aligned} \boldsymbol{\mathcal{D}}^{\parallel}\left(\mathbf{x}, \mathbf{R}_k\right) &= -\sum_k Q_k \nabla \frac{1}{4\pi \left|\mathbf{x} - \mathbf{R}_k\right|} \\ &= \sum_k Q_k \frac{\partial}{\partial \mathbf{R}_k} \frac{1}{4\pi \left|\mathbf{x} - \mathbf{R}_k\right|}. \end{aligned} \tag{SM 21}$$

We can then write the ion position derivative of the Lagrangian as

$$\begin{aligned} \frac{\partial \bar{L}}{\partial \mathbf{R}_k} &= \frac{\partial}{\partial \mathbf{R}_k} \int d^3x \, \left(\frac{\boldsymbol{\mathcal{D}}^2\left(\mathbf{x}, \mathbf{R}_k\right)}{2} + \boldsymbol{\mathcal{D}}\left(\mathbf{x}, \mathbf{R}_k\right) \cdot \boldsymbol{\mathcal{P}}\left(\mathbf{x}, \mathbf{r}\right) \right) \\ &= -\int d^3x \, \left(\mathbf{E}(\mathbf{x}) \cdot \frac{\partial}{\partial \mathbf{R}_k} \right) \boldsymbol{\mathcal{D}}^{\parallel}\left(\mathbf{x}, \mathbf{R}_k\right), \end{aligned} \tag{SM 22}$$

where we have enforced that $\boldsymbol{\mathcal{P}}$ and $\boldsymbol{\mathcal{D}}^{\perp}$ are not functions of $\mathbf{R}_k$ and that $\partial/\partial \mathbf{R}_k \times \boldsymbol{\mathcal{D}}^{\parallel} = 0$ because $\boldsymbol{\mathcal{D}}^{\parallel}$ is a longitudinal field with respect to $\mathbf{R}_k$. The j-th component of the integrand is therefore

$$\left[\left(\mathbf{E}(\mathbf{x}) \cdot \frac{\partial}{\partial \mathbf{R}_k} \right) \boldsymbol{\mathcal{D}}^{\parallel}\left(\mathbf{x}, \mathbf{R}_k\right) \right]_j \; = \; Q_k \left(E_i(\mathbf{x}) \frac{\partial}{\partial R_{k,i}} \right) \frac{\partial}{\partial R_{k,j}} \left(\frac{1}{4\pi \left|\mathbf{x} - \mathbf{R}_k\right|} \right), \tag{SM 23}$$

where the sum over i is implied. The longitudinal and transverse delta functions are [9]

$$\begin{aligned}\delta^{\parallel}_{ij}(\mathbf{x}) &= -\lim_{\epsilon\to 0}\frac{\partial}{\partial x_i}\frac{\partial}{\partial x_j}\frac{1}{4\pi\sqrt{\mathbf{x}^2+\epsilon^2}}\\ &=\frac{1}{3}\delta_{ij}(\mathbf{x})+\frac{1}{4\pi\left|\mathbf{x}\right|^3}\left(\delta_{ij}-3\frac{x_i x_j}{\left|\mathbf{x}\right|^2}\right)\end{aligned} \tag{SM 24}$$

and

$$\delta^{\perp}_{ij}(\mathbf{x}) = \delta_{ij}(\mathbf{x}) - \delta^{\parallel}_{ij}(\mathbf{x}), \tag{SM 25}$$

where δ_{ij} is a Kronecker-delta and $\delta_{ij}(\mathbf{x}) \equiv \delta_{ij}\delta(\mathbf{x})$. Therefore,

$$\left[\left(\mathbf{E}(\mathbf{x})\cdot\frac{\partial}{\partial \mathbf{R}_k}\right)\boldsymbol{\mathcal{D}}^{\parallel}\left(\mathbf{x},\mathbf{R}_k\right)\right]_j = -Q_k E_i(x)\delta^{\parallel}_{ij}\left(\mathbf{x}-\mathbf{R}_k\right), \tag{SM 26}$$

which gives the Lorentz force

$$M_k\ddot{\mathbf{R}}_k = Q_k\mathbf{E}^{\parallel}(\mathbf{R}_k). \tag{SM 27}$$

The lack of magnetic force and transverse electric force originates from assuming that the ions do not generate any current. Therefore, the ions are not affected by magnetic forces and the electrostatic force is conservative which means it has zero curl and so no transverse component.

Since $\dot{\boldsymbol{\mathcal{D}}}^{\parallel} = 0$, the mechanical degree of freedom $\boldsymbol{\mathcal{D}}^{\parallel}$ does not have a canonical momentum. The ion position does, however, have a canonical momentum given by

$$\mathbf{P}_k = \frac{\partial \bar{L}}{\partial \dot{\mathbf{R}}_k} = M_k\dot{\mathbf{R}}_k, \tag{SM 28}$$

which brings an additional kinetic energy term to the total Hamiltonian $\bar{H}$. This Hamiltonian is then found to be (using $\mathbf{V} = V_0 = 0$)

$$\begin{aligned}\bar{H} =& \frac{1}{2m}\left[\mathbf{p}+\mathbf{\Phi}(\mathbf{r})\right]^2+\sum_k\frac{\mathbf{P}_k^2}{2M_k}+\frac{1}{2}\int d^3x\,\left(\frac{1}{\varepsilon_0}\left[\boldsymbol{\mathcal{D}}^{\perp}(\mathbf{x})-\boldsymbol{\mathcal{P}}\left(\mathbf{x},\mathbf{r}\right)\right]^2+\frac{1}{\mu_0}\mathbf{B}^2(\mathbf{x})\right)\\ &+\frac{1}{\varepsilon_0}\int d^3x\,\left(\frac{1}{2}\left[\boldsymbol{\mathcal{D}}^{\parallel}\left(\mathbf{x},\mathbf{R}_k\right)\right]^2+\boldsymbol{\mathcal{D}}^{\parallel}\left(\mathbf{x},\mathbf{R}_k\right)\cdot\boldsymbol{\mathcal{P}}\left(\mathbf{x},\mathbf{r}\right)\right).\end{aligned} \tag{SM 29}$$

As described in the main text, we can quantise the $\mathbf{C}^{\perp}$-field as in Eqn. (25). The radiation fields are quantised exactly as without the ionic lattice. That is, for the $\mathbf{C}^{\perp}$-field quantised as in Eqn. (25), $\boldsymbol{\mathcal{D}}^{\perp}$ takes the form

$$\widehat{\boldsymbol{\mathcal{D}}}^{\perp}(\mathbf{x}) = -i\sum_{\mathbf{k}\lambda}\left(\mathbf{k}\times\boldsymbol{\epsilon}_{\mathbf{k}\lambda}\right)f_{\mathbf{k}}\big(\hat{\mathfrak{c}}^{\dagger}_{\mathbf{k}\lambda}\mathrm{e}^{-i\mathbf{k}\cdot\mathbf{x}}-\hat{\mathfrak{c}}_{\mathbf{k}\lambda}\mathrm{e}^{i\mathbf{k}\cdot\mathbf{x}}\big), \tag{SM 30}$$

and the magnetic field is just the canonical momentum. Therefore, using the canonical commutation relation $\left[\widehat{C}_i(\mathbf{x}),\widehat{B}_j(\mathbf{x}')\right] = \left[\widehat{C}_i(\mathbf{x}),\widehat{B}^{\perp}_j(\mathbf{x}')\right] = i\delta^{\perp}_{ij}(\mathbf{x}-\mathbf{x}')$ where the second equality follows from $\boldsymbol{\nabla}\cdot\mathbf{B} = 0$, we find that

$$\widehat{\mathbf{B}}(\mathbf{x}) = i\sum_{\mathbf{k}\lambda}\nu_{\mathbf{k}}\boldsymbol{\epsilon}_{\mathbf{k}\lambda}f_{\mathbf{k}}\left(\hat{\mathfrak{c}}^{\dagger}_{\mathbf{k}\lambda}\mathrm{e}^{-i\mathbf{k}\cdot\mathbf{x}}-\hat{\mathfrak{c}}_{\mathbf{k}\lambda}\mathrm{e}^{i\mathbf{k}\cdot\mathbf{x}}\right). \tag{SM 31}$$

After quantising the radiation fields in this way, it is clear that the first line of Eqn. (SM 29) will lead to the Hamiltonian describing the dipole interacting with the light field that is given in the main text in Eqn. (26), so long as the magnetic effects in $\mathbf{\Phi}$ are ignored and the electric dipole approximation is made to simplify $\boldsymbol{\mathcal{P}}(\mathbf{x}) \approx -e\mathbf{r}\delta(\mathbf{x})$. Quantising the second line of Eqn. (SM 29) leads to a description of the influence (on the dipole) of vibrations in the ionic lattice from their mean positions. From Eqn. (SM 19) we can see that the total energy of the phonon field is described by the terms

$$\sum_k\frac{\mathbf{P}_k^2}{2M_k}+\int d^3x\,\frac{1}{2\varepsilon_0}\left[\widehat{\boldsymbol{\mathcal{D}}}^{\parallel}\left(\mathbf{x},\mathbf{R}_k\right)\right]^2 = \sum_{\mathbf{q}\mu}\omega_{\mathbf{q}\mu}\left(\hat{b}^{\dagger}_{\mathbf{q}\mu}\hat{b}_{\mathbf{q}\mu}+\frac{1}{2}\right), \tag{SM 32}$$

where $b_{\mathbf{q}\mu}$ ($b^{\dagger}_{\mathbf{q}\mu}$) is the annihilation (creation) operator of a phonon of wavenumber $\mathbf{q}$ and polarisation μ. Details of the derivation of Eqn. (SM 32) can be found in, for example, Refs. [1–3, 10]. The final term in the Hamiltonian, $\int d^3x\ \widehat{\boldsymbol{\mathcal{D}}}^{\parallel} \cdot \widehat{\boldsymbol{\mathcal{P}}}$, describes the interaction between the phonons and the dipole. After linearising this interaction by assuming that the ions are only displaced from equilibrium by small amounts, one can then show that in the EDA [1–3, 10]

$$\frac{1}{\varepsilon_0}\int d^3x\ \widehat{\boldsymbol{\mathcal{D}}}^{\parallel}(\mathbf{x},\mathbf{R}_k)\cdot\widehat{\boldsymbol{\mathcal{P}}}(\mathbf{x},\mathbf{r}) \approx \sum_{\mathbf{q}\mu}\left(\hat{b}^{\dagger}_{\mathbf{q}\mu}+\hat{b}_{\mathbf{q}\mu}\right)\sum_{n=1}^{N} g_{n\mathbf{q}\mu}\left|\epsilon_n\right\rangle\left\langle\epsilon_n\right|, \tag{SM 33}$$

which is the usual phonon interaction encountered throughout the literature where the coupling strength of dipole level n to phonon mode with wavenumber $\mathbf{q}$ and polarisation μ is $g_{n\mathbf{q}\mu}$. We can finally write the full Hamiltonian describing the dynamics of a dipole in an EM field interacting with a vibrating ionic lattice (with $\mathbf{V} = V_0 = 0$):

$$\begin{aligned}\widehat{H} =& \sum_{n=0}^{N}\epsilon_n\left|\epsilon_n\right\rangle\left\langle\epsilon_n\right| - \frac{1}{\varepsilon_0}\hat{\mathbf{d}}\cdot\left(\boldsymbol{\nabla}\times\widehat{\mathbf{C}}^{\perp}(0)\right) + \frac{1}{\varepsilon_0}\sum f^2_{\mathbf{k}}\nu_{\mathbf{k}}\left(\hat{\mathbf{d}}\cdot\boldsymbol{\epsilon}_{\mathbf{k}\boldsymbol{\lambda}}\right)^2 + \sum_{\mathbf{k}\lambda}\nu_{\mathbf{k}}\hat{\mathfrak{c}}^{\dagger}_{\mathbf{k}\boldsymbol{\lambda}}\hat{\mathfrak{c}}_{\mathbf{k}\boldsymbol{\lambda}}\\ &+\sum_{\mathbf{q}\mu}\left(\hat{b}^{\dagger}_{\mathbf{q}\mu}+\hat{b}_{\mathbf{q}\mu}\right)\sum_{n=1}^{N} g_{n\mathbf{q}\mu}\left|\epsilon_n\right\rangle\left\langle\epsilon_n\right| + \sum_{\mathbf{q}\mu}\omega_{\mathbf{q}\mu}\hat{b}^{\dagger}_{\mathbf{q}\mu}\hat{b}_{\mathbf{q}\mu}.\end{aligned} \tag{SM 34}$$

SUPPLEMENTARY MATERIAL 3: DERIVING THE EQUATIONS OF MOTION AND HAMILTONIAN FROM THE $\mathbf{V} = V_0 = 0$ THEORY

In this section, we will derive the equations of motion and the Hamiltonian of the $\mathbf{C}$-field theory from the Lagrangian given in the main text by Eqn. (18). For ease of reading, this Lagrangian is

$$L = \frac{1}{2}m\dot{\mathbf{r}}^2 + \int d^3x\ \frac{1}{2}\left(\mu_0\left[\mathbf{H}(\mathbf{x})+\mathbf{M}(\mathbf{x},\mathbf{r})\right]^2 - \frac{1}{\varepsilon_0}\left[\mathbf{D}(\mathbf{x})-\mathbf{P}(\mathbf{x},\mathbf{r})\right]^2\right),$$

where $\mathbf{D} = \boldsymbol{\nabla}\times\mathbf{C}$ and $\mathbf{H} = \boldsymbol{\nabla}C_0 + \dot{\mathbf{C}}$, which automatically satisfy Gauss's law $\boldsymbol{\nabla}\cdot\mathbf{D} = 0$ and Maxwell-Ampere's law $\boldsymbol{\nabla}\times\mathbf{H} = \dot{\mathbf{D}}$. Note that the equations of motion for the Lagrangian transformed by Eqns. (17) must be identical because the transformation preserves the equations of motion by construction.

A. No magnetic monopoles

The non-existence of magnetic monopoles means that $\mathbf{B}$ is sourceless and hence divergenceless: $\boldsymbol{\nabla}\cdot\mathbf{B} = 0$. This equation of motion is found from the Euler-Lagrange equation

$$\frac{d}{dt}\frac{\partial L}{\partial\dot{C}_0(\mathbf{x})} - \frac{\partial L}{\partial C_0(\mathbf{x})} = 0. \tag{SM 35}$$

Since L does not depend on $\dot{C}_0$ we can immediately write that $\partial L/\partial\dot{C}_0 = 0$. We can also show that

$$\begin{aligned}\frac{\partial L}{\partial C_0(\mathbf{x})} &= \frac{\partial}{\partial C_0(\mathbf{x})}\int d^3x'\ \mu_0\left[\frac{1}{2}\mathbf{H}^2(\mathbf{x}') + \mathbf{H}(\mathbf{x}')\cdot\mathbf{M}(\mathbf{x}',\mathbf{r})\right]\\ &= \int d^3x'\ \mu_0\left[\mathbf{H}(\mathbf{x}')+\mathbf{M}(\mathbf{x}',\mathbf{r})\right]\cdot\frac{\partial}{\partial C_0(\mathbf{x})}\nabla C_0(\mathbf{x}')\\ &= -\int d^3x'\ \left[\boldsymbol{\nabla}\cdot\mathbf{B}(\mathbf{x}')\right]\frac{\partial C_0(\mathbf{x}')}{\partial C_0(\mathbf{x})}\\ &= -\boldsymbol{\nabla}\cdot\mathbf{B}(\mathbf{x}),\end{aligned} \tag{SM 36}$$

where, to arrive at the third line, we have integrated by parts and, to arrive at the final line, we have used that $\partial C_0(\mathbf{x}')/\partial C_0(\mathbf{x}) = \delta(\mathbf{x}-\mathbf{x}')$. This completes the proof. Note that treating C_0 as an independent coordinate results in a singular Hessian and so a non-invertible Lagrangian. It is better to not treat C_0 as a coordinate, and instead impose $\boldsymbol{\nabla}\cdot\mathbf{B} = 0$ as a primary constraint on the Lagrangian. This is identical to Gauss's law $\boldsymbol{\nabla}\cdot\mathbf{E} = \rho$ and A_0 in the conventional theory.

B. Faraday's equation

Faraday's equation, $\boldsymbol{\nabla} \times \mathbf{E} = -\dot{\mathbf{B}}$, is found from the Euler-Lagrange equation

$$\frac{d}{dt}\frac{\partial L}{\partial \dot{\mathbf{C}}(\mathbf{x})} - \frac{\partial L}{\partial \mathbf{C}(\mathbf{x})} = 0. \tag{SM 37}$$

We start with the derivative with respect to $\mathbf{C}$, for which we find that

$$\begin{aligned}\frac{\partial L}{\partial \dot{\mathbf{C}}(\mathbf{x})} &= \frac{\partial}{\partial \dot{\mathbf{C}}(\mathbf{x})}\int d^3x' \; \mu_0 \left[\frac{1}{2}\mathbf{H}^2(\mathbf{x}') + \mathbf{H}(\mathbf{x}')\cdot\mathbf{M}\left(\mathbf{x}',\mathbf{r}\right)\right] \\ &= \int d^3x' \; \mathbf{B}(\mathbf{x}')\cdot\frac{\partial \dot{\mathbf{C}}(\mathbf{x}')}{\partial \dot{\mathbf{C}}(\mathbf{x})} \\ &= \mathbf{B}(\mathbf{x}),\end{aligned} \tag{SM 38}$$

and note that this is also the conjugate momentum of the field. The other derivative is

$$\frac{\partial L}{\partial \mathbf{C}(\mathbf{x})} = \frac{\partial}{\partial \mathbf{C}(\mathbf{x})}\int d^3x' \; \frac{1}{\varepsilon_0}\left[-\frac{1}{2}\mathbf{D}^2(\mathbf{x}') + \mathbf{D}(\mathbf{x}')\cdot\mathbf{P}\left(\mathbf{x}',\mathbf{r}\right)\right]. \tag{SM 39}$$

This is slightly more complicated so we will take each term individually. The $\mathbf{D}^2$ term is evaluated using $\mathbf{D} = \boldsymbol{\nabla}\times\mathbf{C}$ and the identity $\partial/\partial\mathbf{W}\left(\boldsymbol{\nabla}\times\mathbf{W}\right)^2 = 2\boldsymbol{\nabla}\times\boldsymbol{\nabla}\times\mathbf{W}$ for any vector field $\mathbf{W}$. The $\mathbf{D}\cdot\mathbf{P}$ term is evaluated as

$$\begin{aligned}&\frac{\partial}{\partial \mathbf{C}(\mathbf{x})}\int d^3x' \; \mathbf{D}(\mathbf{x}')\cdot\mathbf{P}\left(\mathbf{x}',\mathbf{r}\right) \\ &\quad = \int d^3x' \; \left[\left(\mathbf{P}\left(\mathbf{x}',\mathbf{r}\right)\cdot\frac{\partial}{\partial \mathbf{C}(\mathbf{x})}\right)\mathbf{D}(\mathbf{x}') + \mathbf{P}\left(\mathbf{x}',\mathbf{r}\right)\times\left(\frac{\partial}{\partial \mathbf{C}(\mathbf{x}')}\times\mathbf{D}(\mathbf{x}')\right)\right].\end{aligned} \tag{SM 40}$$

For the first term of Eqn. (SM 40) we find that

$$\left(\mathbf{P}\left(\mathbf{x}',\mathbf{r}\right)\cdot\frac{\partial}{\partial \mathbf{C}(\mathbf{x})}\right)\mathbf{D}(\mathbf{x}') = \boldsymbol{\nabla}\times\left[\mathbf{P}\left(\mathbf{x}',\mathbf{r}\right)\delta(\mathbf{x}-\mathbf{x}')\right]. \tag{SM 41}$$

The second term in Eqn. (SM 40) is zero because $\partial/\partial\mathbf{C}\times\left[\boldsymbol{\nabla}\times\mathbf{C}\right] = \boldsymbol{\nabla}\times\left[\partial/\partial\mathbf{C}\times\mathbf{C}\right] = 0$. Therefore, we find that

$$\begin{aligned}\frac{\partial L}{\partial \mathbf{C}(\mathbf{x})} &= -\boldsymbol{\nabla}\times\int d^3x' \; \frac{1}{\varepsilon_0}\left[\mathbf{D}(\mathbf{x}') - \mathbf{P}\left(\mathbf{x}',\mathbf{r}\right)\right]\delta(\mathbf{x}-\mathbf{x}') \\ &= -\boldsymbol{\nabla}\times\mathbf{E}(\mathbf{x}).\end{aligned} \tag{SM 42}$$

Together, Eqns. (SM 38) and (SM 42) complete the proof.

C. Lorentz force

The proceeding derivation of the Lorentz force is very lengthy. It can be bypassed by noting that our Hamiltonian in Eqn. (23) is identical to the multipolar Hamiltonian in [5] when written in terms of the physical fields. Since the multipolar gauge Hamiltonian is equivalent to the Coulomb gauge Hamiltonian, and it is readily proven that the Coulomb gauge Hamiltonian reproduces the Lorentz force through Heisenberg's equation, so must the $\mathbf{C}$-field Hamiltonian. This argument can only be made for the Lorentz force derivation because this does not depend on the choice of potentials to characterise the physical fields. Nevertheless, for concreteness here we will derive the Lorentz force explicitly from the $\mathbf{C}$-field Lagrangian.

The Lorentz force of the electron, $m\ddot{\mathbf{r}} = -e\left[\mathbf{E}(\mathbf{r}) + \dot{\mathbf{r}}\times\mathbf{B}(\mathbf{r})\right]$, is found from the Euler-Lagrange equation

$$\frac{d}{dt}\frac{\partial L}{\partial \dot{\mathbf{r}}} - \frac{\partial L}{\partial \mathbf{r}} = 0. \tag{SM 43}$$

Note that to derive the Lorentz force for the hole one must redefine the origin of the coordinate system such that the hole is not located on it. Then, subsequently evaluate the Euler-Lagrange with respect to the new position vector of the hole. To evaluate Eqn. (SM 43) consider the velocity derivative,

$$\frac{\partial L}{\partial \dot{\mathbf{r}}} = m\dot{\mathbf{r}} + \frac{\partial}{\partial \dot{\mathbf{r}}}\int d^3x \; \mu_0\left[\mathbf{H}(\mathbf{x})\cdot\mathbf{M}\left(\mathbf{x},\mathbf{r}\right) + \frac{1}{2}\mathbf{M}^2\left(\mathbf{x},\mathbf{r}\right)\right]. \tag{SM 44}$$

It can be shown that

$$\frac{\partial}{\partial\dot{\mathbf{r}}}\mathbf{H}(\mathbf{x})\cdot\mathbf{M}(\mathbf{x},\mathbf{r}) = -\boldsymbol{\theta}(\mathbf{x},\mathbf{r})\times\mathbf{H}(\mathbf{x}), \tag{SM 45}$$

$$\frac{\partial}{\partial\dot{\mathbf{r}}}\frac{1}{2}\mathbf{M}^2(\mathbf{x},\mathbf{r}) = -\boldsymbol{\theta}(\mathbf{x},\mathbf{r})\times\mathbf{M}(\mathbf{x},\mathbf{r})\,. \tag{SM 46}$$

Therefore,

$$\begin{aligned}\frac{\partial L}{\partial\dot{\mathbf{r}}} &= m\dot{\mathbf{r}} - \int d^3x\ \boldsymbol{\theta}(\mathbf{x},\mathbf{r})\times\mathbf{B}(\mathbf{x})\\ &= m\dot{\mathbf{r}} + e\int_0^1 d\lambda\ \lambda\mathbf{r}\times\mathbf{B}(\lambda\mathbf{r}),\end{aligned} \tag{SM 47}$$

which is also equal to the conjugate momentum $\mathbf{p}$ in Eqn. (21a) if $\mathbf{V} = V_0 = 0$. We then take the total time derivative of this to find

$$\frac{\mathrm{d}}{\mathrm{d}t}\frac{\partial L}{\partial\dot{\mathbf{r}}} = m\ddot{\mathbf{r}} + e\int_0^1 d\lambda\ \lambda\Big(\dot{\mathbf{r}}\times\mathbf{B} + \mathbf{r}\times\dot{\mathbf{B}}(\lambda\mathbf{r}) + \mathbf{r}\times\left[(\dot{\mathbf{r}}\cdot\nabla_{\mathbf{r}})\,\mathbf{B}(\lambda\mathbf{r})\right]\Big). \tag{SM 48}$$

The position derivative separates into electric and magnetic parts

$$\frac{\partial L}{\partial\mathbf{r}} = \int d^3x\ \nabla_{\mathbf{r}}\left(\mathcal{L}_{\mathrm{elec}} + \mathcal{L}_{\mathrm{mag}}\right), \tag{SM 49}$$

where

$$\begin{aligned}\nabla_{\mathbf{r}}\mathcal{L}_{\mathrm{elec}} &= \varepsilon_0^{-1}\nabla_{\mathbf{r}}\left(\mathbf{D}(\mathbf{x})\cdot\mathbf{P}(\mathbf{x},\mathbf{r}) - \frac{1}{2}\mathbf{P}^2(\mathbf{x},\mathbf{r})\right)\\ &= \left[\mathbf{E}(\mathbf{x})\cdot\nabla_{\mathbf{r}}\right]\mathbf{P}(\mathbf{x},\mathbf{r}) + \mathbf{E}(\mathbf{x})\times\left[\nabla_{\mathbf{r}}\times\mathbf{P}(\mathbf{x},\mathbf{r})\right]\\ &= \tilde{\nabla}_{\mathbf{P}}\left[\mathbf{E}(\mathbf{x})\cdot\mathbf{P}(\mathbf{x},\mathbf{r})\right],\end{aligned} \tag{SM 50}$$

and the terms involving partial derivatives of $\mathbf{D}$ with respect to $\mathbf{r}$ are zero. To arrive at the last line we have used the identity $\mathbf{a}\times(\nabla_{\mathbf{r}}\times\mathbf{b}) = \tilde{\nabla}_{\mathbf{b}}(\mathbf{a}\cdot\mathbf{b}) - (\mathbf{a}\cdot\nabla_{\mathbf{r}})\,\mathbf{b}$ where the gradient operator with a tilde is the Feynman subscript notation, i.e.

$$\tilde{\nabla}_{\mathbf{b}}(\mathbf{a}\cdot\mathbf{b}) = \left(\mathbf{a}\cdot\frac{\partial\mathbf{b}}{\partial r_x}, \mathbf{a}\cdot\frac{\partial\mathbf{b}}{\partial r_y}, \mathbf{a}\cdot\frac{\partial\mathbf{b}}{\partial r_z}\right). \tag{SM 51}$$

Likewise the magnetic part is

$$\begin{aligned}\nabla_{\mathbf{r}}\mathcal{L}_{\mathrm{mag}} &= \nabla_{\mathbf{r}}\left(\mathbf{H}(\mathbf{x})\cdot\mathbf{M}(\mathbf{x},\mathbf{r}) + \frac{1}{2}\mathbf{M}^2(\mathbf{x},\mathbf{r})\right)\\ &= \left[\mathbf{B}(\mathbf{x})\cdot\nabla_{\mathbf{r}}\right]\mathbf{M}(\mathbf{x},\mathbf{r}) + \mathbf{B}(\mathbf{x})\times\left[\nabla_{\mathbf{r}}\times\mathbf{M}(\mathbf{x},\mathbf{r})\right]\\ &= \tilde{\nabla}_{\mathbf{M}}\left[\mathbf{B}(\mathbf{x})\cdot\mathbf{M}(\mathbf{x},\mathbf{r})\right],\end{aligned} \tag{SM 52}$$

and partial derivatives of $\mathbf{H}$ with respect to $\mathbf{r}$ are zero. The electric part evaluates to

$$\begin{aligned}\int d^3x\ \nabla_{\mathbf{r}}\mathcal{L}_{\mathrm{elec}} &= \int d^3x\ \tilde{\nabla}_{\mathbf{P}}\left[\mathbf{E}(\mathbf{x})\cdot\mathbf{P}(\mathbf{x},\mathbf{r})\right]\\ &= -e\int_0^1 d\lambda\ \int d^3x\ \Big[\mathbf{E}(\mathbf{x}) + \left(\mathbf{E}(\mathbf{x})\cdot\mathbf{r}\right)\nabla_{\mathbf{r}}\Big]\delta(\mathbf{x}-\lambda\mathbf{r})\,.\end{aligned} \tag{SM 53}$$

and the magnetic part is

$$\begin{aligned}\int d^3x\ \nabla_{\mathbf{r}}\mathcal{L}_{\mathrm{mag}} &= \int d^3x\ \tilde{\nabla}_{\mathbf{M}}\left[\mathbf{B}(\mathbf{x})\cdot\mathbf{M}(\mathbf{x},\mathbf{r})\right]\\ &= -e\int_0^1 d\lambda\ \int d^3x\ \Big\{\dot{\mathbf{r}}\times\mathbf{B}(\mathbf{x}) + \left(\mathbf{r}\cdot\left[\dot{\mathbf{r}}\times\mathbf{B}(\mathbf{x})\right]\right)\nabla_{\mathbf{r}}\Big\}\delta(\mathbf{x}-\lambda\mathbf{r})\,.\end{aligned} \tag{SM 54}$$

Before substituting into the Euler-Lagrange equation we note that by using Faraday's law we can rewrite the second term in Eqn. (SM 48) as

$$\begin{aligned}
e\int_0^1 d\lambda\ \lambda\mathbf{r}\times\dot{\mathbf{B}}(\lambda\mathbf{r}) &= -e\int_0^1 d\lambda\ \mathbf{r}\times[\nabla_{\mathbf{r}}\times\mathbf{E}(\lambda\mathbf{r})] \\
&= -e\int_0^1 d\lambda\ \Big\{\tilde{\nabla}_{\mathbf{E}}[\mathbf{r}\cdot\mathbf{E}(\lambda\mathbf{r})]-[\mathbf{r}\cdot\nabla_{\mathbf{r}}]\mathbf{E}(\lambda\mathbf{r})\Big\} \\
&= -e\int_0^1 d\lambda\ \int d^3x\ \Big\{[\mathbf{r}\cdot\mathbf{E}(\mathbf{x})]\nabla_{\mathbf{r}}-\mathbf{E}(\mathbf{x})[\mathbf{r}\cdot\nabla_{\mathbf{r}}]\Big\}\delta(\mathbf{x}-\lambda\mathbf{r}),
\end{aligned} \tag{SM 55}$$

where to arrive at the last line we have written $\mathbf{E}(\lambda\mathbf{r})=\int d^3x\ \mathbf{E}(\mathbf{x})\delta(\mathbf{x}-\lambda\mathbf{r})$. Substituting Eqns. (SM 48), (SM 53), (SM 54) and (SM 55) into the Euler-Lagrange equation results in

$$\begin{aligned}
m\ddot{\mathbf{r}} = &-e\int_0^1 d\lambda\ \int d^3x\ \Big\{\mathbf{E}(\mathbf{x})[1+(\mathbf{r}\cdot\nabla_{\mathbf{r}})] \\
&+\lambda\Big[[\mathbf{r}\times\mathbf{B}(\mathbf{x})](\dot{\mathbf{r}}\cdot\nabla_{\mathbf{r}})+2\dot{\mathbf{r}}\times\mathbf{B}+(\mathbf{r}\cdot[\dot{\mathbf{r}}\times\mathbf{B}(\mathbf{x})])\nabla_{\mathbf{r}}\Big]\Big\}\delta(\mathbf{x}-\lambda\mathbf{r}),
\end{aligned} \tag{SM 56}$$

where we have also written $\mathbf{B}(\lambda\mathbf{r})=\int d^3x\ \mathbf{B}(\mathbf{x})\delta(\mathbf{x}-\lambda\mathbf{r})$. After some algebra we can then prove that

$$[\mathbf{r}\times\mathbf{B}(\mathbf{x})](\dot{\mathbf{r}}\cdot\nabla_{\mathbf{r}})+(\mathbf{r}\cdot[\dot{\mathbf{r}}\times\mathbf{B}(\mathbf{x})])\nabla_{\mathbf{r}}=[\dot{\mathbf{r}}\times\mathbf{B}(\mathbf{x})](\mathbf{r}\cdot\nabla_{\mathbf{r}})+(\mathbf{r}\times\dot{\mathbf{r}})[\mathbf{B}(\mathbf{x})\cdot\nabla_{\mathbf{r}}], \tag{SM 57}$$

which when substituted into Eqn. (SM 56) along with $\nabla_{\mathbf{r}}\delta(\mathbf{x}-\lambda\mathbf{r})=-\lambda\nabla_{\mathbf{x}}\delta(\mathbf{x}-\lambda\mathbf{r})$ we find

$$\begin{aligned}
m\ddot{\mathbf{r}} = &-e\int d^3x\ \mathbf{E}(\mathbf{x})\int_0^1 d\lambda\ [1-\lambda(\mathbf{r}\cdot\nabla_{\mathbf{x}})]\delta(\mathbf{x}-\lambda\mathbf{r}) \\
&-e\int d^3x\ \ [\dot{\mathbf{r}}\times\mathbf{B}(\mathbf{x})]\int_0^1 d\lambda\ \lambda(2-\lambda\mathbf{r}\cdot\nabla_{\mathbf{x}})\delta(\mathbf{x}-\lambda\mathbf{r}) \\
&-\int_0^1 d\lambda\ \lambda^2(\mathbf{r}\times\dot{\mathbf{r}})\int d^3x\ \ [\mathbf{B}(\mathbf{x})\cdot\nabla_{\mathbf{x}}]\delta(\mathbf{x}-\lambda\mathbf{r}).
\end{aligned}$$

From here the Lorentz force is derived by noting that because of the non-existence of magnetic monopoles

$$\int d^3x\ \ [\mathbf{B}(\mathbf{x})\cdot\nabla_{\mathbf{x}}]\delta(\mathbf{x}-\lambda\mathbf{r})=-\int d^3x\ \ [\nabla_{\mathbf{x}}\cdot\mathbf{B}(\mathbf{x})]\delta(\mathbf{x}-\lambda\mathbf{r})=0, \tag{SM 58}$$

and that the following identities hold

$$\int_0^1 d\lambda\ \ (1-\lambda\mathbf{r}\cdot\nabla_{\mathbf{x}})\delta(\mathbf{x}-\lambda\mathbf{r})=\delta(\mathbf{x}-\mathbf{r}), \tag{SM 59}$$

$$\int_0^1 d\lambda\ \lambda(2-\lambda\mathbf{r}\cdot\nabla_{\mathbf{x}})\delta(\mathbf{x}-\lambda\mathbf{r})=\delta(\mathbf{x}-\mathbf{r}). \tag{SM 60}$$

These identities can be proven using the Fourier representation of the delta function [5]. Using these we find that $m\ddot{\mathbf{r}}=-e[\mathbf{E}(\mathbf{r})+\dot{\mathbf{r}}\times\mathbf{B}(\mathbf{r})]$ as required.

D. Hamiltonian

The Hamiltonian is derived as is standard by

$$H=\sum_i \mathbf{p}_i\cdot\dot{\mathbf{q}}_i-L, \tag{SM 61}$$

where $\mathbf{q}_i$ and $\mathbf{p}_i$ are the generalised coordinates and canonical momenta. For this theory, the canonical momenta of the matter and $\mathbf{C}$-field are given in Eqns. (21) with $\mathbf{V}=V_0=0$. We find that

$$H=\mathbf{p}\cdot\dot{\mathbf{r}}-\frac{1}{2}m\dot{\mathbf{r}}^2+\int d^3x\ \Big(\mathbf{B}(\mathbf{x})\cdot\dot{\mathbf{C}}(\mathbf{x})-\frac{\mu_0}{2}\mathbf{B}^2(\mathbf{x})+\frac{\varepsilon_0}{2}\mathbf{E}^2(\mathbf{x})\Big). \tag{SM 62}$$

Focusing first on the terms within the integral, we can rewrite $\mathbf{B}\cdot\dot{\mathbf{C}} = \mathbf{B}\cdot\mathbf{H} - \mathbf{B}\cdot\nabla C_0$. The term $\mathbf{B}\cdot\nabla C_0$ then vanishes after integration by parts and enforcing $\boldsymbol{\nabla}\cdot\mathbf{B} = 0$. Since the canonical field momentum is $\mathbf{B}$, we want to eliminate $\mathbf{H}$ in favour of $\mathbf{B}$ to make the quantisation process easier. Therefore, writing $\mathbf{H}+\mathbf{M} = \mathbf{B}/\mu_0$ we arrive at

$$H = \mathbf{p}\cdot\dot{\mathbf{r}} - \frac{1}{2}m\dot{\mathbf{r}}^2 + \frac{1}{2}\int d^3x\ \left(\varepsilon_0\mathbf{E}^2(\mathbf{x}) + \frac{1}{\mu_0}\mathbf{B}^2(\mathbf{x}) - 2\mathbf{B}(\mathbf{x})\cdot\mathbf{M}\left(\mathbf{x},\mathbf{r}\right)\right). \tag{SM 63}$$

Using the definitions of $\boldsymbol{\theta}$ and $\boldsymbol{\Phi}_0$ from the main text we are then able to show that

$$\int d^3x\ \mathbf{B}(\mathbf{x})\cdot\mathbf{M}\left(\mathbf{x},\mathbf{r}\right) = -\frac{1}{m}\left[\mathbf{p}\cdot\boldsymbol{\Phi}_0(\mathbf{r}) + \boldsymbol{\Phi}_0^2(\mathbf{r})\right]. \tag{SM 64}$$

Finally, after combining this with the terms outside the integral using $m\dot{\mathbf{r}} = \mathbf{p}+\boldsymbol{\Phi}_0$, we have

$$H = \frac{1}{2m}\left[\mathbf{p}+\boldsymbol{\Phi}_0(\mathbf{r})\right]^2 + \int d^3x\ \left(\frac{\mathbf{B}^2(\mathbf{x})}{2\mu_0} + \frac{1}{2\varepsilon_0}\left[\mathbf{D}^{\perp}(\mathbf{x}) - \mathbf{P}\left(\mathbf{x},\mathbf{r}\right)\right]^2\right), \tag{SM 65}$$

as in Eqn. (23) of the main text. Note that here we have explicitly used that the longitudinal component of $\mathbf{D}$ is zero because there is no free charge. To align with the Hamiltonians in Supplementary Material 1 for the conventional theory we will ignore the magnetic interactions governed by $\boldsymbol{\Phi}_0$. Quantising the radiation fields is described in Supplementary Material 2 and results in Eqn. (26) in the main text.

SUPPLEMENTARY MATERIAL 4: DERIVING THE CANONICAL MOMENTA AND HAMILTONIAN WITH NONZERO V AND V_0

A. Field and matter canonical momenta

After transformation by Eqns. (17) the Lagrangian is

$$\widetilde{L} = \frac{1}{2}m\dot{\mathbf{r}}^2 + \int d^3x\ \widetilde{\mathcal{L}}(\mathbf{x}), \tag{SM 66}$$

where

$$\widetilde{\mathcal{L}}(\mathbf{x}) = \frac{1}{2}\left(\frac{1}{\mu_0}\mathbf{B}^2(\mathbf{x}) - \varepsilon_0\mathbf{E}^2(\mathbf{x})\right) \tag{SM 67}$$

$$= \frac{1}{2}\left(\mu_0\left[\widetilde{\mathbf{H}}(\mathbf{x}) + \widetilde{\mathbf{M}}\left(\mathbf{x},\mathbf{r}\right)\right]^2 - \frac{1}{\varepsilon_0}\left[\widetilde{\mathbf{D}}(\mathbf{x}) - \widetilde{\mathbf{P}}\left(\mathbf{x},\mathbf{r}\right)\right]^2\right). \tag{SM 68}$$

Also, recall the definitions $\widetilde{\mathbf{P}} = \mathbf{P}+\widetilde{\mathbf{P}}_V$, $\widetilde{\mathbf{M}} = \mathbf{M}-\widetilde{\mathbf{M}}_V$,

$$\widetilde{\mathbf{P}}_V\left(\mathbf{x},\mathbf{r}\right) = \boldsymbol{\nabla}\times\mathbf{V}\left(\mathbf{x},\mathbf{r}\right), \tag{SM 69}$$

$$\widetilde{\mathbf{M}}_V\left(\mathbf{x},\mathbf{r}\right) = \dot{\mathbf{V}}\left(\mathbf{x},\mathbf{r}\right) + \boldsymbol{\nabla}V_0\left(\mathbf{x},\mathbf{r}\right), \tag{SM 70}$$

$$\widetilde{\mathbf{D}}(\mathbf{x}) = \boldsymbol{\nabla}\times\widetilde{\mathbf{C}}(\mathbf{x}), \tag{SM 71}$$

$$\widetilde{\mathbf{H}}(\mathbf{x}) = \dot{\widetilde{\mathbf{C}}}(\mathbf{x}) + \boldsymbol{\nabla}\widetilde{C}_0(\mathbf{x}), \tag{SM 72}$$

where we have written the implicit change in $\mathbf{C}$ (C_0) due to the change in light/matter partition explicitly as $\mathbf{C}\to\widetilde{\mathbf{C}}$ ($C_0\to\widetilde{C}_0$). It is clear that the field canonical momentum will not change from the $\mathbf{V} = V_0 = 0$ case, because it is equal to the physical field $\mathbf{B}$. Therefore we trivially find that

$$\widetilde{\boldsymbol{\Pi}}(\mathbf{x}) = \frac{\delta\widetilde{\mathcal{L}}}{\delta\dot{\widetilde{\mathbf{C}}}(\mathbf{x})} = \mu_0\left[\widetilde{\mathbf{H}}(\mathbf{x}) + \widetilde{\mathbf{M}}\left(\mathbf{x},\mathbf{r}\right)\right] = \mathbf{B}(\mathbf{x}). \tag{SM 73}$$

For the matter canonical momentum we must evaluate

$$\widetilde{\mathbf{p}} = m\dot{\mathbf{r}} + \mu_0\boldsymbol{\nabla}_{\dot{\mathbf{r}}}\int d^3x\ \left(\widetilde{\mathbf{H}}(\mathbf{x})\cdot\widetilde{\mathbf{M}}\left(\mathbf{x},\mathbf{r}\right) + \frac{1}{2}\widetilde{\mathbf{M}}^2\left(\mathbf{x},\mathbf{r}\right)\right). \tag{SM 74}$$

To do so we recall some algebraic relations used in the $\mathbf{V} = V_0 = 0$ case:

$$\nabla_{\dot{\mathbf{r}}}\left[\mathbf{W}(\mathbf{x}) \cdot \mathbf{M}(\mathbf{x}, \mathbf{r})\right] = -\boldsymbol{\theta}(\mathbf{x}, \mathbf{r}) \cdot \mathbf{W}(\mathbf{x}) \;\forall\; \mathbf{W}(\mathbf{x}), \tag{SM 75a}$$

$$\nabla_{\dot{\mathbf{r}}} \frac{1}{2} \mathbf{M}^2(\mathbf{x}, \mathbf{r}) = -\boldsymbol{\theta}(\mathbf{x}, \mathbf{r}) \cdot \mathbf{M}(\mathbf{x}, \mathbf{r}). \tag{SM 75b}$$

We can expand the integral as

$$\widetilde{\mathbf{p}} = m\dot{\mathbf{r}} + \mu_0 \nabla_{\dot{\mathbf{r}}} \int d^3x \left\{ \frac{1}{2}\mathbf{M}^2(\mathbf{x}) + \frac{1}{2}\widetilde{\mathbf{M}}_V^2(\mathbf{x}, \mathbf{r}) - \widetilde{\mathbf{H}}(\mathbf{x}) \cdot \mathbf{M}(\mathbf{x}, \mathbf{r}) - \left[\mathbf{M}(\mathbf{x}, \mathbf{r}) + \widetilde{\mathbf{H}}(\mathbf{x})\right] \cdot \widetilde{\mathbf{M}}_V(\mathbf{x}, \mathbf{r}) \right\}. \tag{SM 76}$$

Using Eqns. (SM 75) we can show that

$$\mu_0 \nabla_{\dot{\mathbf{r}}} \int d^3x \left(\frac{1}{2}\mathbf{M}^2(\mathbf{x}, \mathbf{r}) - \widetilde{\mathbf{H}}(\mathbf{x}) \cdot \mathbf{M}(\mathbf{x}, \mathbf{r}) \right) = -\mu_0 \int d^3x\; \boldsymbol{\theta}(\mathbf{r}) \times \left[\mathbf{M}(\mathbf{x}, \mathbf{r}) + \widetilde{\mathbf{H}}(\mathbf{x})\right]. \tag{SM 77}$$

Then, using standard vector calculus identities we find that the remaining terms can be written as

$$\begin{aligned} \mu_0 \nabla_{\dot{\mathbf{r}}} \int d^3x & \left(\frac{1}{2}\widetilde{\mathbf{M}}_V^2(\mathbf{x}, \mathbf{r}) - \left[\mathbf{M}(\mathbf{x}, \mathbf{r}) + \widetilde{\mathbf{H}}(\mathbf{x})\right] \cdot \widetilde{\mathbf{M}}_V(\mathbf{x}, \mathbf{r}) \right) \\ &= \int d^3x \left(\mu_0 \left\{ \left[\widetilde{\mathbf{M}}_V \cdot \nabla_{\dot{\mathbf{r}}}\right] \mathbf{M} + \widetilde{\mathbf{M}}_V \times \left[\nabla_{\dot{\mathbf{r}}} \times \mathbf{M}\right] \right\} \right. \\ &\qquad \left. - \left\{ \left[\mathbf{B} \cdot \nabla_{\dot{\mathbf{r}}}\right] \widetilde{\mathbf{M}}_V + \mathbf{B} \times \left[\nabla_{\dot{\mathbf{r}}} \times \widetilde{\mathbf{M}}_V\right] \right\} \right) \end{aligned} \tag{SM 78}$$

$$= \int d^3x \left\{ \mu_0 \left[\boldsymbol{\theta}(\mathbf{x}, \mathbf{r}) \times \widetilde{\mathbf{M}}_V(\mathbf{x}, \mathbf{r})\right] - \nabla_{\dot{\mathbf{r}}} \left[\mathbf{B}(\mathbf{x}) \cdot \widetilde{\mathbf{M}}_V(\mathbf{x}, \mathbf{r})\right] \right\}. \tag{SM 79}$$

Combining these, we find that

$$\widetilde{\mathbf{p}} = m\dot{\mathbf{r}} - \int d^3x\; \boldsymbol{\theta}(\mathbf{r}) \times \mathbf{B}(\mathbf{x}) - \nabla_{\dot{\mathbf{r}}} \int d^3x\; \mathbf{B}(\mathbf{x}) \cdot \widetilde{\mathbf{M}}_V(\mathbf{x}, \mathbf{r}) \tag{SM 80}$$

$$\equiv m\dot{\mathbf{r}} - \boldsymbol{\Phi}_0(\mathbf{r}) - \widetilde{\boldsymbol{\Phi}}(\mathbf{r}) \tag{SM 81}$$

as in Eqn. (21a) in the main text.

B. Hamiltonian

As usual we write the Hamiltonian in terms of the coordinates and momenta as in Eqn. (SM 61)

$$\widetilde{H} = \widetilde{\mathbf{p}} \cdot \dot{\mathbf{r}} - \frac{1}{2}m\dot{\mathbf{r}}^2 + \int d^3x \left(\mathbf{B}(\mathbf{x}) \cdot \dot{\widetilde{\mathbf{C}}}(\mathbf{x}) - \frac{1}{2\mu_0}\mathbf{B}^2(\mathbf{x}) + \frac{\varepsilon_0}{2}\mathbf{E}^2(\mathbf{x}) \right). \tag{SM 82}$$

We then define $\widetilde{\mathbf{X}} = \boldsymbol{\Phi}_0 + \widetilde{\boldsymbol{\Phi}}$ and note the following three relations:

- Using the definition $\dot{\widetilde{\mathbf{C}}} + \nabla \widetilde{C}_0 = \mu_0^{-1}\mathbf{B} - \widetilde{\mathbf{M}}$ and that $\nabla \cdot \mathbf{B} = 0$ leads to

$$\int d^3x\; \mathbf{B}(\mathbf{x}) \cdot \dot{\widetilde{\mathbf{C}}}(\mathbf{x}) = \int d^3x \left(\frac{1}{\mu_0}\mathbf{B}^2(\mathbf{x}) - \mathbf{B}(\mathbf{x}) \cdot \widetilde{\mathbf{M}}(\mathbf{x}, \mathbf{r}) \right). \tag{SM 83}$$

- The terms outside the volume integral can be written as

$$\widetilde{\mathbf{p}} \cdot \dot{\mathbf{r}} - \frac{1}{2}m\dot{\mathbf{r}}^2 = \frac{1}{2m}\left(\widetilde{\mathbf{p}}^2 - \widetilde{\mathbf{X}}^2(\mathbf{r})\right). \tag{SM 84}$$

- We can rewrite

$$-\int d^3x\; \mathbf{B}(\mathbf{x}) \cdot \mathbf{M}(\mathbf{x}, \mathbf{r}) = \frac{1}{m}\left(\widetilde{\mathbf{p}} \cdot \widetilde{\mathbf{X}}(\mathbf{r}) + \widetilde{\mathbf{X}}^2(\mathbf{r})\right) - \dot{\mathbf{r}} \cdot \widetilde{\boldsymbol{\Phi}}(\mathbf{r}). \tag{SM 85}$$

Inserting these three relations into Eqn. (SM 82) leads to

$$\widetilde{H} = \frac{1}{2m}\left(\widetilde{\mathbf{p}} + \widetilde{\mathbf{X}}(\mathbf{r})\right)^2 + \frac{1}{2}\int d^3x \, \left[\frac{1}{\mu_0}\mathbf{B}^2(\mathbf{x}) + \varepsilon_0\mathbf{E}^2(\mathbf{x})\right] + (1 - \dot{\mathbf{r}}\cdot\boldsymbol{\nabla}_{\dot{\mathbf{r}}})\int d^3x \; \mathbf{B}(\mathbf{x})\cdot\widetilde{\mathbf{M}}_V\left(\mathbf{x},\mathbf{r}\right). \tag{SM 86}$$

The third term in Eqn. (SM 86) is a result of not being able to invert the matter canonical momentum for an expression for $\dot{\mathbf{r}}$ in terms of $\widetilde{\mathbf{p}}$. We therefore impose a constraint on the choice of $\mathbf{V}$ and V_0 such that:

$$(1 - \dot{\mathbf{r}}\cdot\boldsymbol{\nabla}_{\dot{\mathbf{r}}})\int d^3x \; \mathbf{B}(\mathbf{x})\cdot\widetilde{\mathbf{M}}_V\left(\mathbf{x},\mathbf{r}\right) = 0. \tag{SM 87}$$

This extra constraint on the transformation follows from

$$\widetilde{\boldsymbol{\Phi}}(\mathbf{r}) = \frac{\partial}{\partial\dot{\mathbf{r}}}\int d^3x \; \mathbf{B}(\mathbf{x})\cdot\widetilde{\mathbf{M}}_V\left(\mathbf{x},\mathbf{r}\right), \tag{SM 88}$$

being in general a complicated function of $\dot{\mathbf{r}}$. Therefore, it is not possible obtain for $\dot{\mathbf{r}}$ in terms of $\widetilde{\mathbf{p}}$ by inverting the canonical momentum Eqn. (21a). As an example of an allowed transformation, consider $\mathbf{V} = \frac{1}{2}\mathbf{r}\times\mathbf{x}$ and $V_0 = 0$. For this transformation, we find that $\widetilde{\boldsymbol{\Phi}} = \frac{1}{2}\int d^3x \; \mathbf{x}\times\mathbf{B}$ which means that we can write $\dot{\mathbf{r}}$ as a function of $\widetilde{\mathbf{p}}$ by inverting Eqn. (21a). Importantly, this also means that the last term of Eqn. (SM 86) vanishes, and we arrive at Eqn. (23) in the main text.

SUPPLEMENTARY MATERIAL 5: TABLE OF EXPERIMENTAL DATA POINTS

TABLE SM 1. Table showing the ranges of values used in Figures 2 with corresponding references. Much of the data rely on estimated parameters and so should be used as guide values. The data range with the asterisk has a much larger maximum value than expected, so should be taken with caution.

Experiment	η	f/eV	Dipole size / μm	g/ν	g/ω_{10}	References
Rb gas in optical cavity	2.0×10^{-4}	0.0045 –0.19	1.6×10^{-4}	2.48×10^{-10} -1.42×10^{-6}	2.48×10^{-10} -1.42×10^{-6}	[11–14]
Quantum dot arrays	0.012 −0.052	0.023 −0.034	0.01 −0.05	5.3×10^{-4} −0.0020	6×10^{-4} −0.0019	[15–19]
Superconducting circuit	5.6×10^{-4} -5.9×10^{-4}	8×10^{-4} −0.029	21.4 −60	0.073 −1.3	0.040 -15.3^*	[20–24]
Rare earth spins in microwave resonator	7.2×10^{-4}	3.3×10^{-4}	299.4	0.15	0.051	[25]
Exciton polaritons	0.0091 −0.0098	0.11 −0.51	0.0070 −0.0076	0.0012 −0.0060	0.0012 −0.0060	[26, 27]
Exciton polaritons in dyes	6.0×10^{-4} −0.0011	44.94 −484.5	5×10^{-4} -5.1×10^{-4}	0.035 −0.037	0.043 −0.30	[28–31]
Intersubband polaritons in quantum wells	3.0×10^{-4} −0.0037	4.7 −98.6	0.0075 −0.032	0.023 −2.8	0.032 −4.0	[32–36]
Electron cyclotron resonances	1.7×10^{-6} -2.0×10^{-4}	0.76 −25.7	0.073 −0.19	0.17 −4.8	0.058 −1.4	[37–40]

We have used various estimates when the dipole size has not been reported. In particular:

- The dipole size x_{10} is approximately a couple of Bohr radii a_0 for atomic species, whereas the effective Bohr radius $a_{\mathrm{eff}} \simeq 1\ \mu$m is used for Rydberg atoms.
- For quantum dots and quantum wells, the physical extent of the device is a good estimate for the dipole size.
- The estimates become slightly more complicated for superconducting circuits, but an approximate value can be extracted in two different ways, depending on the values reported: First from the definition of the dipole moment

$$x_{10} \simeq |\mathbf{d}|/e \text{ with } |\mathbf{d}| = \hbar g/E_{\mathrm{vac}}, \tag{SM 89}$$

$$\Rightarrow x_{10} \simeq \hbar g/(eE_{\mathrm{vac}}), \tag{SM 90}$$

where g is the interaction strength, and E_{vac} is the electric field amplitude of the vacuum fluctuations. Electric field fluctuations can in turn be estimated by $E_{\text{vac}} \simeq (V_{\text{vac}}/\text{length of relevant region})$, with V_{vac} being the fluctuations in the electric potential. Alternatively, it is possibly to define an analogous Bohr radius a_{eff} through the resonance frequency, as in

$$\omega_{10} = 2\pi c/\lambda_{10} = 2\pi c \left(\frac{\alpha}{4\pi a_{\text{eff}}} \right), \tag{SM 91}$$

$$\Rightarrow a_{\text{eff}} \simeq \frac{\alpha c}{2\omega_{10}}, \tag{SM 92}$$

where α is the fine-structure constant and where we have interpreted the transition wavelength $\lambda_{10} = 4\pi a_{\text{eff}}/\alpha$ as an effective Bohr wavelength.

- In dye-filled polariton cavities, the spatial extent of the dye molecule acts as a bound of the dipole size.
- In the case of electron cyclotron resonances, we can estimate the dipole size as

$$x_{10} \simeq l_0\sqrt{\nu}, \tag{SM 93}$$

where $l_0 = \sqrt{\hbar/(eB)}$ is the magnetic length for a given magnetic field strength B, and $\nu = \rho_{\text{2deg}} 2\pi l_0^2$ is the filling factor in the system with ρ_{2deg} being the electron density. Note that in some cases, the filling factor is directly reported.

SUPPLEMENTARY MATERIAL 6: ACCURACY OF FEW LEVEL TRUNCATION WITHOUT RESONANCE

Figure SM 1 is the same calculation as for Figure 2 in the main text, however, the splitting between the two lowest lying dipole levels is no longer kept resonant with the single photon mode ν. Instead, we fix the length of the infinite square well such that the lowest lying dipole transition, $\omega_{10} = \epsilon_1 - \epsilon_0 = 1$ eV is held constant. Outwith resonance, the light-matter coupling ratios with ω_{10} and ν in either gauge is not the same, and so the contours along the diagonal-axis and x-axis are different. Just as in Figure 2, even without resonance the **C**-field calculations yield more accurate results for a given coupling strength than the Coulomb gauge, and also converge onto the exact answer quicker as the number of dipole levels in the calculation is increased. Note also that it is for large dipoles in the strong-coupling limit that the **C**-field calculations become inaccurate, i.e. in the scenario when we expect the description of the polarisation field in terms of dipole degrees of freedom to become non-trivial.

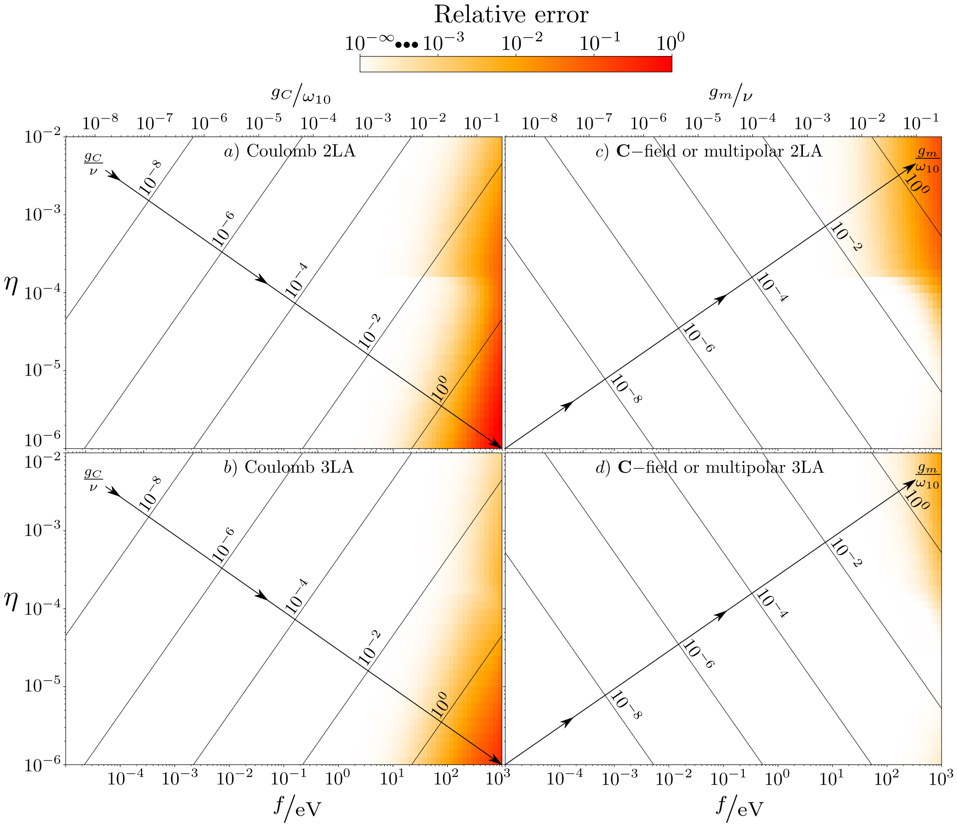

FIG. SM 1. As in Figure 2 in the main text, we plot the relative error in calculating the energy spacing between the two lowest eigenstates of the full Coulomb gauge and **C**-field Hamiltonians within the two and three dipole-level approximations, using an infinite square well potential as $\widehat{U}_{\mathrm{ext}}$. The difference is that in this plot the lowest lying dipole transition is kept fixed at 1 eV, and so, is not on resonance with the mode frequency which is varied along the y-axes. For all other details refer to Figure **??**.

[1] H. Bruus and K. Flensberg, *Many-body quantum theory in condensed matter physics: an introduction* (Oxford university press, 2004).
[2] P. Kok and B. W. Lovett, *Introduction to optical quantum information processing* (Cambridge University Press, 2010).
[3] G. D. Mahan, *Many-particle physics* (Springer Science & Business Media, 2013).
[4] A. Stokes and A. Nazir, Nature communications **10**, 499 (2019).
[5] M. Babiker and R. Loudon, Proceedings of the Royal Society of London. A. Mathematical and Physical Sciences **385**, 439 (1983).
[6] E. Rousseau and D. Felbacq, Scientific reports **7**, 11115 (2017).
[7] D. De Bernardis, T. Jaako, and P. Rabl, Physical Review A **97**, 043820 (2018).
[8] D. De Bernardis, P. Pilar, T. Jaako, S. De Liberato, and P. Rabl, Physical Review A **98**, 053819 (2018).
[9] A. Stewart, arXiv preprint arXiv:0801.0335 (2008).
[10] A. Nazir and D. P. McCutcheon, Journal of Physics: Condensed Matter **28**, 103002 (2016).
[11] A. Suleymanzade, A. Anferov, M. Stone, R. K. Naik, J. Simon, and D. Schuster, arXiv preprint arXiv:1911.00553 (2019).
[12] Y. Colombe, T. Steinmetz, G. Dubois, F. Linke, D. Hunger, and J. Reichel, Nature **450**, 272 (2007).
[13] J. D. Thompson, T. Tiecke, N. P. de Leon, J. Feist, A. Akimov, M. Gullans, A. S. Zibrov, V. Vuletić, and M. D. Lukin, Science **340**, 1202 (2013).
[14] T. Tiecke, J. D. Thompson, N. P. de Leon, L. Liu, V. Vuletić, and M. D. Lukin, Nature **508**, 241 (2014).
[15] J. Gérard, B. Sermage, B. Gayral, B. Legrand, E. Costard, and V. Thierry-Mieg, Physical review letters **81**, 1110 (1998).
[16] J. Gerard, D. Barrier, J. Marzin, R. Kuszelewicz, L. Manin, E. Costard, V. Thierry-Mieg, and T. Rivera, Applied Physics Letters **69**, 449 (1996).
[17] J. Gerard, B. Legrand, B. Gayral, E. Costard, B. Sermage, R. Kuszelewicz, D. Barrier, V. Thierry-Mieg, T. Rivera, and J. Marzin, Physica E: Low-dimensional Systems and Nanostructures **2**, 804 (1998).
[18] B. Gayral, J. Gérard, A. Lemaıtre, C. Dupuis, L. Manin, and J. Pelouard, Applied physics letters **75**, 1908 (1999).
[19] E. Moreau, I. Robert, J. Gérard, I. Abram, L. Manin, and V. Thierry-Mieg, Applied Physics Letters **79**, 2865 (2001).
[20] J. Johansson, S. Saito, T. Meno, H. Nakano, M. Ueda, K. Semba, and H. Takayanagi, Physical Review Letters **96**, 127006 (2006).
[21] T. Niemczyk, F. Deppe, H. Huebl, E. Menzel, F. Hocke, M. Schwarz, J. Garcia-Ripoll, D. Zueco, T. Hümmer, E. Solano, *et al.*, Nature Physics **6**, 772 (2010).
[22] P. Forn-Díaz, J. Lisenfeld, D. Marcos, J. J. Garcia-Ripoll, E. Solano, C. Harmans, and J. Mooij, Physical review letters **105**, 237001 (2010).
[23] A. Baust, E. Hoffmann, M. Haeberlein, M. Schwarz, P. Eder, J. Goetz, F. Wulschner, E. Xie, L. Zhong, F. Quijandría, *et al.*, Physical Review B **93**, 214501 (2016).
[24] F. Yoshihara, T. Fuse, S. Ashhab, K. Kakuyanagi, S. Saito, and K. Semba, Nature Physics **13**, 44 (2017).
[25] J. Everts, G. G. King, N. Lambert, S. Kocsis, S. Rogge, and J. J. Longdell, arXiv preprint arXiv:1911.11311 (2019).
[26] C. Weisbuch, M. Nishioka, A. Ishikawa, and Y. Arakawa, Physical Review Letters **69**, 3314 (1992).
[27] J. Bloch, T. Freixanet, J. Marzin, V. Thierry-Mieg, and R. Planel, Applied physics letters **73**, 1694 (1998).
[28] J. Bellessa, C. Bonnand, J. Plenet, and J. Mugnier, Physical review letters **93**, 036404 (2004).
[29] H.-S. Wei, C.-C. Jaing, Y.-T. Chen, C.-C. Lin, C.-W. Cheng, C.-H. Chan, C.-C. Lee, and J.-F. Chang, Optics express **21**, 21365 (2013).
[30] S. Gambino, M. Mazzeo, A. Genco, O. Di Stefano, S. Savasta, S. Patanè, D. Ballarini, F. Mangione, G. Lerario, D. Sanvitto, *et al.*, ACS Photonics **1**, 1042 (2014).
[31] S. Kéna-Cohen, S. A. Maier, and D. D. Bradley, Advanced Optical Materials **1**, 827 (2013).
[32] E. Dupont, H. Liu, A. SpringThorpe, W. Lai, and M. Extavour, Physical Review B **68**, 245320 (2003).
[33] E. Dupont, J. Gupta, and H. Liu, Physical Review B **75**, 205325 (2007).
[34] Y. Todorov, A. M. Andrews, R. Colombelli, S. De Liberato, C. Ciuti, P. Klang, G. Strasser, and C. Sirtori, Physical review letters **105**, 196402 (2010).
[35] A. Delteil, A. Vasanelli, Y. Todorov, C. F. Palma, M. R. St-Jean, G. Beaudoin, I. Sagnes, and C. Sirtori, Physical review letters **109**, 246808 (2012).
[36] B. Askenazi, A. Vasanelli, A. Delteil, Y. Todorov, L. Andreani, G. Beaudoin, I. Sagnes, and C. Sirtori, New Journal of Physics **16**, 043029 (2014).
[37] V. Muravev, I. Andreev, I. Kukushkin, S. Schmult, and W. Dietsche, Physical Review B **83**, 075309 (2011).
[38] G. Scalari, C. Maissen, D. Turčinková, D. Hagenmüller, S. De Liberato, C. Ciuti, C. Reichl, D. Schuh, W. Wegscheider, M. Beck, *et al.*, Science **335**, 1323 (2012).
[39] C. Maissen, G. Scalari, F. Valmorra, M. Beck, J. Faist, S. Cibella, R. Leoni, C. Reichl, C. Charpentier, and W. Wegscheider, Physical Review B **90**, 205309 (2014).
[40] A. Bayer, M. Pozimski, S. Schambeck, D. Schuh, R. Huber, D. Bougeard, and C. Lange, Nano letters **17**, 6340 (2017).
